# Combining advanced radiotherapy techniques and immunotherapy: immunomodulatory mechanisms and clinical prospects

**DOI:** 10.3389/fimmu.2026.1737661

**Published:** 2026-02-18

**Authors:** Linsen Zhou, Qianyi Liu, Yongze He, Ying Tang, Shuotong Liu, Xiaobin Wang, Shiyan Shen, Jialin Ji, Zhen Liu, Xianhu Zeng, Jiangping Li

**Affiliations:** 1 Department of Radiotherapy Physics & Technology, Cancer Center, West China Hospital, Sichuan University, Chengdu, Sichuan, China; 2 West China School of Medicine, Sichuan University, Chengdu, Sichuan, China; 3 Division of Thoracic Tumor Multimodality Treatment, Cancer Center, West China Hospital, Sichuan University, Chengdu, Sichuan, China

**Keywords:** carbon ion radiotherapy, FLASH radiotherapy, immune checkpoint inhibitors, immunotherapy, proton therapy, SBRT, SFRT, tumor microenvironment

## Abstract

This review systematically explores the mechanisms and clinical prospects of combining advanced radiotherapy techniques—including stereotactic body radiotherapy, FLASH radiotherapy, proton therapy, carbon ion radiotherapy, and spatially fractionated radiotherapy—with immunotherapy. It elucidates how radiotherapy activates systemic antitumor immunity by inducing immunogenic cell death, activating the cGAS–STING pathway, upregulating MHC-I expression, and remodeling the tumor microenvironment. These mechanisms synergize with immune checkpoint inhibitors to enhance both local and systemic tumor control, including the abscopal effect. Compared with conventional radiotherapy, these advanced techniques leverage precise dose distributions, ultra-high dose rates, or the Bragg Peak physical properties to better protect normal tissues, mitigate radiation-induced lymphopenia, and favorably modulate the tumor microenvironment. Although preclinical studies and emerging clinical trials support the feasibility and efficacy of such combinations, further large-scale, well-designed clinical trials are required to validate their optimal application strategies and long-term benefits.

## 1 Introduction

Radiation therapy, as a critical component of cancer treatment, exhibits diverse mechanisms of action. Classical radiobiology attributes the clinical efficacy of radiotherapy to DNA damage caused by direct and indirect effects of radiation-generated reactive oxygen species, leading to failure in cell division or the accumulation of lethal damage. With advances in research, it has been discovered that radiotherapy can also modulate both local and systemic immune responses (1). Factors such as the tumor microenvironment play a crucial role in determining the overall response of tumors to radiotherapy (2). With the advancement of radiotherapy technologies, the emergence of novel techniques such as stereotactic body radiotherapy (SBRT), FLASH radiotherapy (FLASH RT), proton therapy, heavy-ion radiotherapy, and spatially fractionated radiotherapy (SFRT) has provided diverse options for cancer treatment and opened new avenues for combining radiotherapy with immunotherapy.

SBRT originated from the concept of stereotactic radiosurgery (SRS) proposed in 1951 by the Swedish neurosurgeon Lars Leksell (3). Its core principle lies in focusing radiation from three-dimensional space to deliver a high dose to the target volume, thereby achieving protection of the surrounding normal tissues (4). With advancements in linear accelerators, image-guided systems, and radiotherapy planning systems, the application of SBRT has been promoted in the treatment of various malignancies such as non-small cell lung cancer (NSCLC) (5), hepatocellular carcinoma (HCC) (6), pancreatic cancer (7, 8), and bone metastases (9).

In 1946, American physicist Robert Wilson first proposed utilizing the physical properties of the proton’s Bragg Peak for cancer treatment (10). In 1954, Lawrence Berkeley Laboratory in the United States conducted the first proton therapy on a breast cancer patient, confirming its clinical feasibility. Building upon the foundation of proton therapy, the concept of heavy ion therapy, specifically CIRT, was further developed. In 1975, the Lawrence Berkeley Laboratory in the United States first treated patients using neon ions (11). CIRT demonstrates particularly unique capabilities in treating tumors that exhibit resistance to conventional radiotherapy and chemotherapy, such as melanoma (12), renal cell carcinoma (13), cervical adenocarcinoma (14), and pancreatic cancer (15).

FLASH RT represents a revolutionary breakthrough in cancer radiation therapy, characterized by ultra-high dose rate (UHDR) irradiation, typically exceeding 40 Gy/s. The FLASH effect was first observed in 1959 by Dewey and his team (16). In 2014, researchers at the Institut Curie in France were the first to systematically elucidate the FLASH effect through animal experiments (17). In 2019, FLASH RT was first applied in a human case (18). With in-depth research, FLASH RT has been explored with various particle types, including electron beams (19), photon beams (20), proton beams (21), and heavy ion beams (22).

SFRT treats the entire tumor with a non-uniform dose distribution. Its core principle lies in utilizing a grid collimator or a multi-leaf collimator (MLC) to segment the radiation field into multiple micro-beams. This creates simultaneous high-dose “peaks” and low-dose “valleys” within the target volume (23). The initial SFRT technique, known as grid therapy, was proposed by Kohler in 1909 (24). Relevant radiobiological studies indicate that the mechanisms of SFRT involve radiation-induced bystander effects (25), microvascular alterations (26) and immune modulation (27). Current indications include bulky or locally advanced diseases unsuitable for conventional radiation or those proven refractory to chemoradiation (**Table 1**).

**Table 1:** Comparison of key characteristics and applications of different radiotherapy techniques.

Characteristics	SBRT	FLASH RT	Proton therapy	Carbon ion therapy	SFRT
Time of clinical application​	1980s	2019	1954	1975	1990s
Types of particles​	Photon	Photon, Proton, Electron, Carbon ion	Proton	Carbon ion	Photon
Core technical principles​	​​Three-dimensional spatial focusing, hypofractionated irradiation.	Ultra-high dose rate (UHDR) irradiation (>40 Gy/s)​.	The Bragg Peak is precisely directed to the tumor site. ​​The dose deposited before the peak is low​​, while ​​beyond the peak, the dose drops to nearly zero.​​	The Bragg Peak of carbon ion beams is sharper and exhibits higher biological effectiveness.	The radiation field is segmented into multiple micro-beams, creating a simultaneous distribution of high-dose ‘peaks’ and low-dose ‘valleys’ within the target volume.
Relative biological effectiveness (RBE)	RBE=1	Depending on the particle types.	RBE=1.1	RBE=2.0~3.5	RBE=1
Indications	Lung cancer, liver cancer, prostate cancer, etc.	Superficial tumors (electron), pain relief from bone metastases (proton).	Pediatric tumors and tumors adjacent to critical organs (brain, spinal cord, eyes)​.	Tumors resistant to photon/proton therapy, such as osteosarcoma, chordoma, and adenoid cystic carcinoma.​	Bulky or locally advanced tumors refractory to chemoradiotherapy.​
Key advantages	Precision, hypofractionation, and shortened treatment courses; with relatively accessible equipment and high cost-effectiveness.​	The FLASH effect can significantly reduce side effects and protect normal tissues.	Significantly reduces the radiation dose to critical organs surrounding the target volume.​	Significantly reduces the radiation dose to critical organs surrounding the target volume, and is more effective against tumors resistant to photon/proton therapy.​	Disperse hot spots within the target volume, reduce the volume of tissue receiving high-dose irradiation, and spare a significant amount of surrounding normal tissue.​

**Figure 1:**
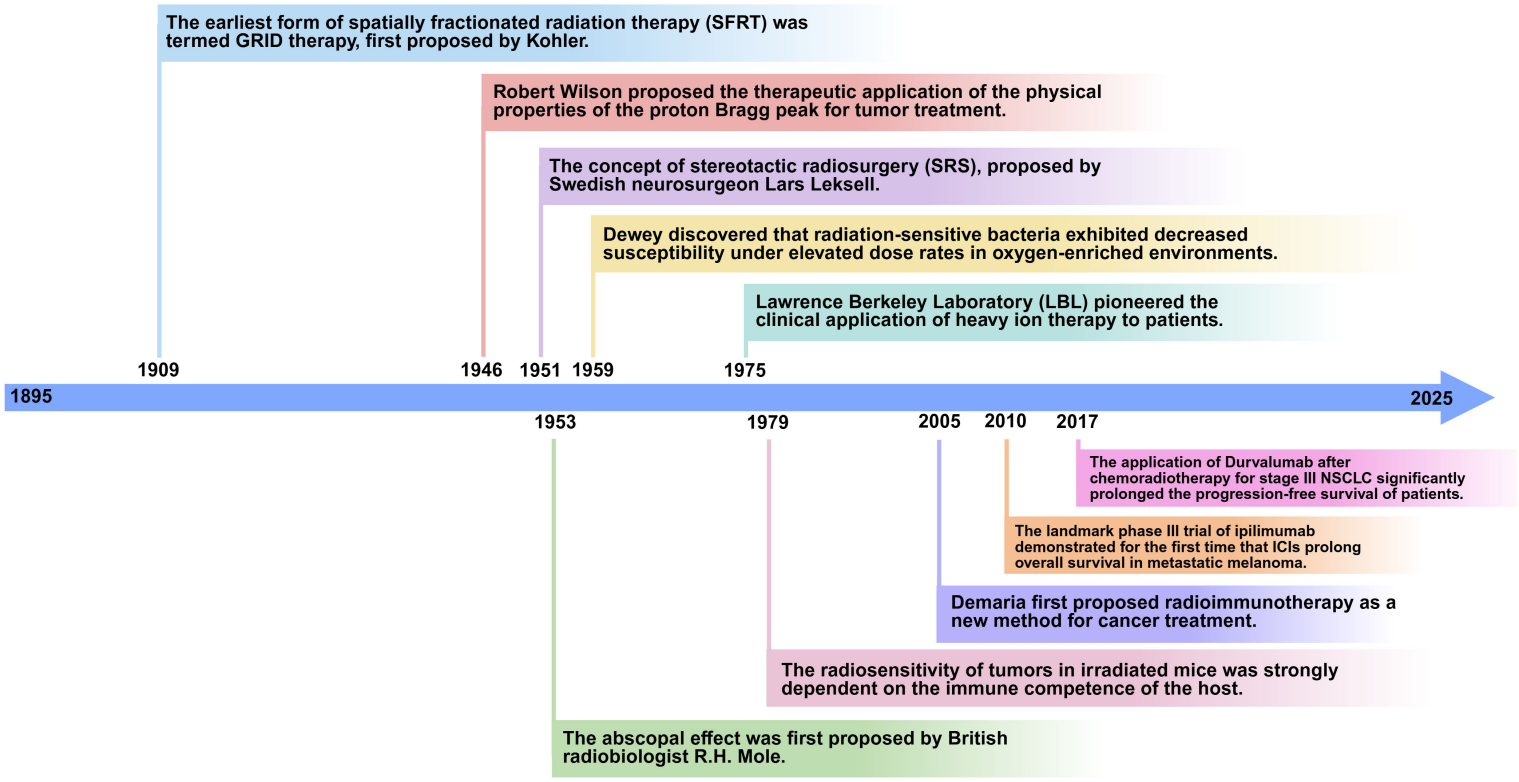
The history of research on different radiotherapy technologies and immunomodory effects. The points above the timeline correspond to the initial proposal dates of various radiation therapy techniques, while the points below the timeline correspond to the key research milestones in the study of immunomodulatory effects associated with radiation therapy.

Parallel to the ongoing development of these radiotherapy techniques, investigations into radiation-induced immunomodulation have been progressively expanding. In 1953, the British radiobiologist R.H. Mole first proposed the concept of the “abscopal effect,” revealing that local radiotherapy can not only effectively target tumors within the irradiated field but also elicit systemic antitumor responses through certain mechanisms (28). In 1973, Ehlers and Fridman first reported a case of the abscopal effect in a solid tumor: when a patient with papillary adenocarcinoma received irradiation to the entire neck and supraclavicular lymph nodes using Cobalt-60, a significant regression of the patient’s mediastinal tumor was observed (29). In 1979, a preclinical study demonstrated that the radiation sensitivity of irradiated murine fibrosarcoma was strongly correlated with the host’s immune activity. The dose required to achieve 50% tumor control in immunocompromised mice was twice that required in immunocompetent mice (30). In 2005, Demaria et al. discussed the experimental evidence for radiotherapy as a tool to induce antitumor immunity and, for the first time, proposed radioimmunotherapy as a novel approach for cancer treatment (31). In 2010, a Phase III trial of ipilimumab demonstrated that the median overall survival (OS) of patients with metastatic melanoma increased from 6.4 months to 10.1 months. This was the first confirmation that an immune checkpoint inhibitors (ICIs) could prolong survival in advanced patients, providing an immunological foundation for its combination with radiotherapy (32). In 2018, a study demonstrated that, compared to placebo, durvalumab significantly prolonged overall survival in patients with unresectable stage III NSCLC who had not progressed after concurrent chemoradiotherapy (33) (**Figure 1**).

This review is divided into four sections: 1) We delineated the mechanisms underlying the immunomodulatory effects of radiotherapy, encompassing both immune-protective and immune-suppressive effects; 2) We elaborated on the immune-protective mechanisms of the aforementioned radiotherapy techniques; 3) We reviewed clinical data on radiotherapy combined with immunotherapy to analyze indications, dosing, efficacy, and safety profiles; 4) We summarized the commonalities in the immunomodulatory mechanisms across different radiotherapy techniques, aiming to inform clinical decision-making for various radiotherapy technologies and their combination with immunotherapy, as well as to guide future clinical trials.

## 2 Mechanistic insights into radiation-induced immunomodulatory effects

The immunomodulatory effects of radiotherapy exhibit a dual nature: it can enhance the host’s anti-tumor immune response but may also induce immunosuppressive effects under certain circumstances. The following sections will elaborate on the mechanisms associated with its immune-activating and immune-suppressive effects, respectively.

### 2.1 The positive immunostimulatory effects of radiotherapy

#### 2.1.1 Radiotherapy upregulates the expression of major histocompatibility complex class I on tumor cells, thereby enhancing the immunogenic visibility of tumor antigens

MHC-I is a protein complex expressed on the cell surface, whose core function is to present endogenous antigenic peptides to cytotoxic T cells, thereby initiating specific immune-mediated killing (34). To evade immune surveillance, tumor cells frequently downregulate MHC-I expression (35). In contrast, radiation can increase the cell surface expression of MHC-I in a dose-dependent manner (36). After a period of irradiation, the mammalian target of rapamycin kinase signaling pathway is activated, enhancing overall protein synthesis. This leads to an increase in intracellular peptide levels and promotes the assembly of MHC-I molecules (36). Upregulation of MHC-I on the tumor cell surface can enhance the infiltration of CD4^+^ and CD8^+^ T lymphocytes and their antigen recognition of tumor cells, thereby improving the host immune system’s capacity to identify and eliminate tumor cells (37). Furthermore, radiotherapy can indirectly upregulate MHC-I expression on the tumor cell surface by inducing the release of interferons. These interferons bind to interferon receptors on the tumor cells, stimulating the phosphorylation of Janus kinases JAK1 and JAK2. This, in turn, leads to the phosphorylation of STAT1. The phosphorylated STAT1 translocates into the nucleus, where it drives the transcription of NLRC5 and IRF1, these transcription factors further stimulates the transcription of MHC-I genes (38).

#### 2.1.2 Radiotherapy activates the cGAS-STING pathway

The stimulator of interferon genes (STING) is an adaptor protein anchored on the endoplasmic reticulum membrane that regulates innate immune signaling transduction. Cyclic GMP-AMP Synthase (cGAS) is a nucleotidyltransferase that senses cytosolic DNA and activates the STING-TBK1-IRF-3 signaling axis, thereby inducing the production of type I interferons (IFN-I) (39). Following irradiation, cells release double-stranded DNA (dsDNA) and mitochondrial DNA (mtDNA), which activates the cGAS-STING signaling pathway (40). cGAS recognizes cytosolic dsDNA and mtDNA, undergoes conformational changes and oligomerization, thereby activating its enzymatic activity. The activated cGAS utilizes intracellular adenosine triphosphate (ATP) and GTP as substrates to catalyze the synthesis of the second messenger 2’,3’-cyclic guanosine monophosphate–adenosine monophosphate (2’,3’-cGAMP). cGAMP binds to STING on the endoplasmic reticulum membrane, inducing its activation. The activated STING translocates from the endoplasmic reticulum to the Golgi apparatus, where it recruits and activates TBK1. TBK1 subsequently phosphorylates the transcription factor IRF3 (41),The phosphorylated IRF3 dimerizes and translocates into the nucleus, inducing the expression of IFN-I and interferon-stimulated genes (ISGs) (42). IFN-I upregulates the expression of MHC-I molecules and activates dendritic cells (DCs), enhancing their antigen presentation capacity (43).It also activates the effector functions of CD8^+^ T cells, thereby augmenting anti-tumor immune responses (44). Furthermore, IFN-I induces the upregulation of CCR7, MIP-3β, and Th1 chemokines, which enhances lymphocyte homing to lymph nodes (45). Furthermore, STING signaling can interact with the NF-κB signaling pathway, further promoting the production of pro-inflammatory cytokine and synergistically enhancing anti-tumor immune responses (42).

#### 2.1.3 Radiotherapy increases the release of damage-associated molecular patterns and induces immunogenic cell death

Radiation therapy can increase the release of damage-associated molecular patterns (DAMPs), which elicits immunogenic cell death (ICD) in tumor cells. DAMPs are a class of endogenous molecules, including calreticulin (CALR), extracellular ATP, and high-mobility group box 1 protein (HMGB1), among others. These molecules are released into the intercellular space or bloodstream upon cellular damage, stress, or death. They promote the recruitment and maturation of DCs, cross-presentation of tumor antigens, and activation of effector T cells (46).

CALR is an endoplasmic reticulum (ER)-resident protein involved in numerous biological processes, including facilitating correct protein folding within the ER, regulating calcium homeostasis, and ensuring proper antigen presentation by MHC-I molecules (47). Radiotherapy can induce endoplasmic reticulum stress through the generation of oxidative stressors, such as reactive oxygen species, leading to the translocation of calreticulin to the surface of tumor cells (48). This surface-exposed CALR serves as an “eat-me” signal for DCs by binding to the low-density lipoprotein receptor-related protein, thereby promoting antigen uptake and presentation, which subsequently activates adaptive anti-tumor immune responses (49).

High Mobility Group Box 1 is a non-histone chromosomal protein widely present in eukaryotic cells. Under physiological conditions, HMGB1 is primarily localized within the nucleus. However, upon cellular exposure to radiation damage, stress, or under pathological conditions, HMGB1 can be translocated and released into the extracellular space (50). HMGB1 can bind to Toll-like receptor 4 (TLR4) and the receptor for advanced glycation end products (RAGE), activating downstream signaling pathways such as NF-κB and MAPK. This activation promotes the secretion of various pro-inflammatory cytokines and enhances the uptake and presentation of tumor antigens by DCs, thereby activating adaptive immunity (51, 52).

In healthy tissues, the extracellular ATP level is extremely low. However, when tumor cells undergo ICD, the autophagy-dependent secretion of ATP serves as a critical DAMP (53). High concentrations of ATP activate the P2X7 receptor on the surface of DCs, inducing conformational changes and opening of its ion channel. This results in K^+^ efflux and Ca²^+^ influx, which activates the NLRP3 inflammasome and facilitates the assembly of a complete NLRP3 inflammasome complex (54). This complex then converts pro-caspase-1 into active caspase-1, which specifically cleaves intracellular pro-IL-1β and pro-IL-18 into their bioactive mature forms. These mature cytokines are subsequently secreted extracellularly to exert their pro-inflammatory effects (55). These cytokines play crucial roles in promoting the activation and proliferation of immune cells.

Furthermore, the release of DAMPs helps create an immunogenic microenvironment within the tumor locale. This facilitates the efficient uptake, processing, and presentation of tumor-associated antigens (TAAs), which are released by dying tumor cells, by DCs. Consequently, this process activates antigen-specific T cells in the lymph nodes. These activated T cells then circulate systemically, recognizing and eliminating primary and metastatic tumors expressing the same TAAs. The immune response is further amplified through antigen spreading (56).

#### 2.1.4 Regulating the tumor microenvironment and promoting the release of various inflammatory factors

The tumor microenvironment is an intricate and complex system composed of diverse cellular and molecular components, including cancer cells, immune cells, fibroblasts, endothelial cells, and the extracellular matrix, among others (57). Radiotherapy can modulate the immune cells and inflammatory mediators within the tumor microenvironment through various mechanisms, thereby remodeling the tumor microenvironment and indirectly influencing tumor growth and metastasis (58). In the tumor microenvironment, macrophages can polarize into distinct functional phenotypes. M1-type macrophages express pro-inflammatory cytokines which promote immune-mediated tumor killing. In contrast, M2-type macrophages express anti-inflammatory cytokines which contribute to the depletion of extracellular L-arginine and drive T cell suppression (59). Research by Klug et al. demonstrated that low-dose radiotherapy (≤2 Gy) can reprogram tumor-associated macrophages towards an M1 phenotype. This repolarization promotes the effective recruitment of tumor-specific T cells and facilitates T cell-mediated tumor rejection (60). Radiotherapy can stimulate tumor cells and stromal cells within the tumor microenvironment to release various cytokines, which influence the migration and function of immune cells. It induces the release of chemokines such as CXCL9, CXCL10, CXCL11, and CXCL16, leading to the recruitment of macrophages, DCs, and T cells to the tumor site (61). Radiation also promotes the release of CXCL8 from tumor cells in an NF-κB-dependent manner, which induces the migration of peripheral blood natural killer (NK) cells to the irradiated tumor sites, thereby enhancing NK cell-mediated tumor cell killing (62). Radiotherapy exerts enabling effects on stromal cells within the tumor microenvironment, while simultaneously inducing direct pro-inflammatory effects on tumor cells themselves. These actions collectively contribute to the establishment of an immune-mediated, tumor-suppressive microenvironment.

#### 2.1.5 The abscopal effect mediated by combined radiotherapy and immunotherapy

The abscopal effect refers to the phenomenon where localized radiotherapy directed at one tumor lesion leads to the shrinkage or disappearance of distant, non-irradiated metastatic lesions. This phenomenon demonstrates that radiotherapy not only contributes to the local control of the targeted lesion but can also elicit a systemic anti-tumor response through certain mechanisms, thereby controlling metastases located far from the treatment site (63).

The occurrence of the abscopal effect involves the complex activation of the immune system, alterations in the tumor microenvironment, and interactions among various molecular and cellular signals (64). It can be regarded as a manifestation of the combined action of the aforementioned radioprotective immune responses. In essence, abscopal effect represents a systemic anti-tumor immune cascade triggered by localized radiotherapy: irradiation induces immunogenic cell death in cancer cells, leading to the exposure of calreticulin and the release of ATP and HMGB1. These DAMPs bind to the CD91, P2RX7, and TLR4 receptors on antigen-presenting cells, respectively, promoting the recruitment of DCs to the tumor site, enhancing their phagocytosis of tumor antigens, and facilitating antigen presentation to T cells (65). TLR4 activation initiates the MyD88-dependent signaling pathway, leading to the nuclear translocation of the transcription factor NF-κB. This process upregulates the expression of MHC-I on DCs and promotes their maturation. Collectively, these mechanisms trigger a robust inflammatory cytokine response, enhance DC maturation, and induce the upregulation of chemokine receptors, thereby driving the migration of DCs to the draining lymph nodes (66). In lymph nodes, mature DCs present tumor antigen peptides to the T-cell receptor via MHC molecules. The binding of CD80/CD86 to CD28 on T cells triggers the production of cytokines, including interleukin-2, which drives T cell proliferation. Simultaneously, intercellular adhesion molecule-1 (ICAM-1) expressed on DCs binds to lymphocyte function-associated antigen-1 on T cells, providing an additional co-stimulatory signal (67). Ultimately, the antigen-activated effector T cells exit the lymph nodes and patrol the body in search of tumor antigens. They can home to both the irradiated tumor and non-irradiated tumors, leading to the regression of distant lesions and manifesting the abscopal effect.

Furthermore, radiotherapy acts on tumor-draining lymph nodes (TDLNs), eliminating local immunosuppressive cells and recruiting newly activated immune cells, thereby enhancing systemic immune surveillance (68). Ultimately, combining with ICIs to block inhibitory pathways such as programmed cell death protein 1 (PD-1) and programmed death-ligand 1 (PD-L1) significantly enhances the activity of cytotoxic T lymphocytes (CTLs), synergistically amplifying the systemic anti-tumor response (69, 70). In recent years, the abscopal effect has been extensively documented across various tumors, including cervical cancer (71), melanoma (72), hepatocellular carcinoma (73), and myeloma (74).

### 2.2 The negative immunosuppressive effects of radiotherapy

#### 2.2.1 Radiation-induced lymphopenia

Lymphocytes are the primary mediators of cell-mediated immunity and play a critical role in promoting systemic immune responses against tumors. Studies have shown that lymphocytes are highly sensitive to radiation, with an LD_50_ (dose required to kill 50% of lymphocytes) of 2 Gy and an LD_90_ (dose required to kill 90% of lymphocytes) of 3 Gy (75, 76). Lymphocytes are susceptible to depletion even at low radiation doses (<1 Gy) (77). Radiation-induced lymphopenia primarily results from the irradiation of lymphatic organs and circulating lymphocytes in the blood. The reduction in absolute lymphocyte count in the blood is associated with factors such as the total radiation dose, the total number of lymphocytes irradiated, and the baseline lymphocyte count (78, 79).

The central lymphoid organs primarily include the bone marrow and the thymus. Radiotherapy can induce bone marrow dysfunction. Since hematopoietic stem cells in the bone marrow are highly sensitive to ionizing radiation (80), even relatively low doses may lead to temporary functional suppression of the bone marrow, while higher radiotherapy doses can cause irreversible damage to hematopoietic function (81). The extent of lymphocyte recovery post-radiotherapy is also largely dependent on the physiological function of the individual patient’s bone marrow. Relevant studies also indicate that radiation therapy not only has a direct impact on irradiated lymphocytes but also exerts indirect effects on lymphocytes and stem cells within non-irradiated bone marrow (82, 83). Furthermore, radiation can induce acute thymic injury and loss of cellular substance (84). Irradiation of the thymus with 1 Gy can lead to an 89% reduction in both CD4^+^ T cells and CD8^+^ T cells (85). Radiotherapy can also directly kill lymphocytes circulating in the blood, adversely affecting immune responses by reducing the numbers of CD4^+^ T and CD8^+^ T cells in the systemic circulation. Research suggests that the post-irradiation decrease in T lymphocyte levels is influenced by lymphocytes within major blood vessels (86). Irradiation of peripheral lymphoid organs can also trigger lymphopenia. Naïve T cells within lymph nodes are sensitive to radiation, and even low-dose irradiation of lymphoid organs can result in rapid, p53-mediated apoptosis. Studies have shown that the risk of developing Grade 3 or higher lymphopenia is associated with the maximum radiation dose delivered to the spleen (87).

#### 2.2.2 Radiation therapy chronically activates the cGAS-STING pathway to exert immunosuppressive effects

As mentioned previously, the release of dsDNA and mtDNA by radiotherapy activates the cGAS-STING signaling pathway, which is a key mechanism for inducing anti-tumor immunity. However, studies have also shown that the activation of the cGAS-STING signaling pathway can lead to immunosuppression through various avenues. The core factor lies in the dual regulatory function of interferons (88). Benci et al. discovered that persistent IFN-I signaling in tumor cells activates the STAT1 transcription factor, leading to sustained high expression of PD-L1 on the tumor cell surface (89). Furthermore, IFN-γ can also upregulate the expression of PD-L1 through the JAK1-STAT1 signaling pathway (90). PD-L1, by binding to the PD-1 receptor on the surface of T cells, inhibits T cell activation, proliferation, and cytokine production, thereby enhancing tumor cell tolerance to self-antigens (91). A study by Du et al. revealed that radiation-induced double-stranded DNA fragments trigger the activation of the cGAS-STING pathway, leading to the upregulation of PD-L1 (92). Furthermore, oxidized mitochondrial DNA can also induce interferon signaling via the cGAS-STING pathway, upregulating PD-L1 and indoleamine 2,3-dioxygenase 1 (IDO-1) expression, thereby suppressing T-cell activation (93). In HCC, impaired BRCA1 function leads to the accumulation of DNA damage, which activates the cGAS-STING pathway and subsequently induces PD-L1 expression, thereby protecting cancer cells from T-cell infiltration (94). Furthermore, the activation of cGAS-STING-mediated type I interferon signaling induces substantial production of the regulatory cytokine IL-10, which enhances the immunosuppressive activity of regulatory T cells (Tregs). Additionally, this signaling promotes the expression of chemokines CCL2, CCL7, and CCL12, thereby recruiting myeloid-derived suppressor cells (MDSCs) to the tumor site (95).

**Figure 2:**
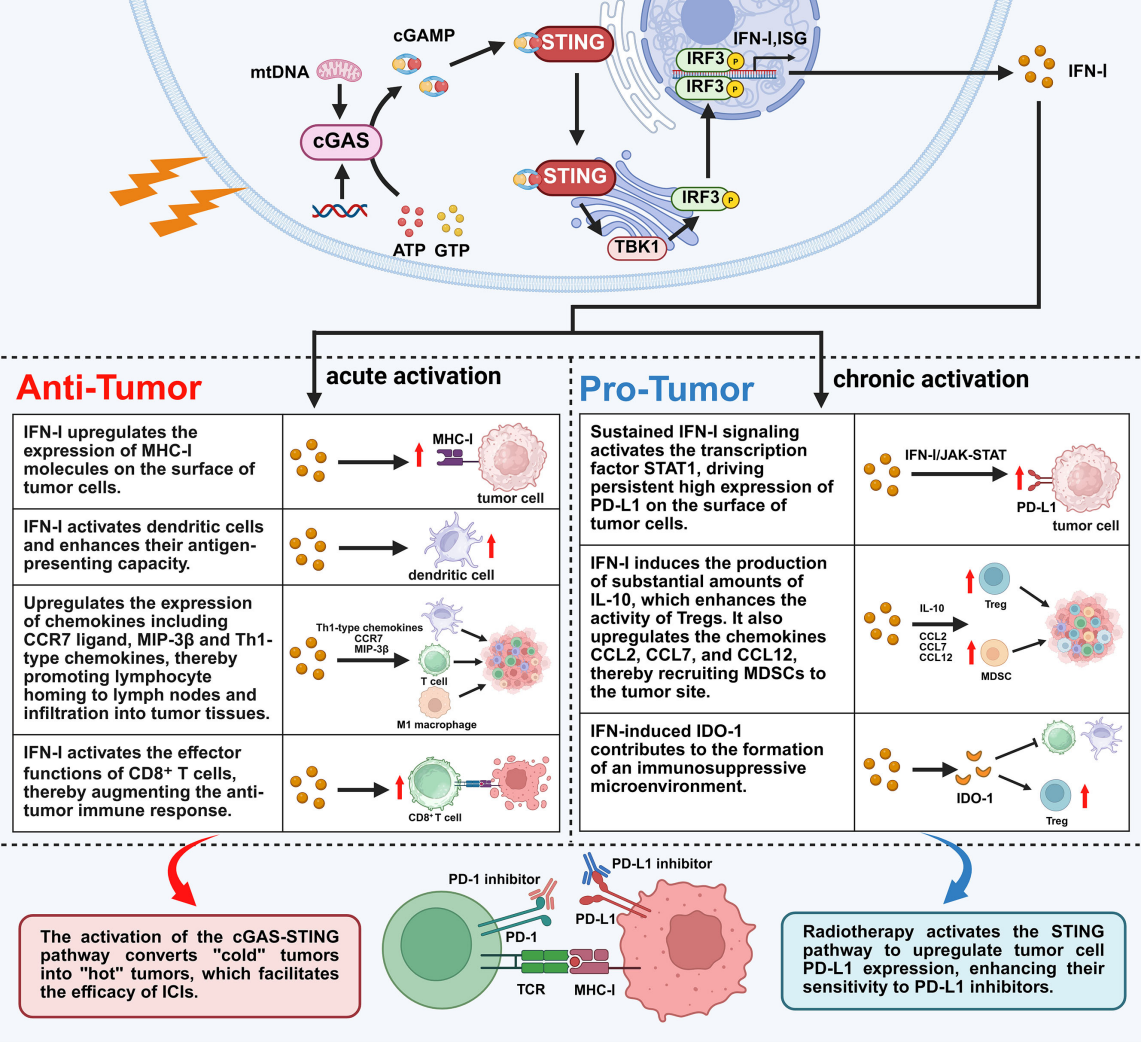
Bidirectional immune effects of cGAS-STING signaling pathway activation and its implications for immunotherapy. The cGAS-STING pathway serves as the common initial trigger. Based on the distinct activation patterns of STING, it exerts bidirectional effects: Left side – Acute STING activation promotes anti-tumor immune responses by upregulating MHC-I, activating DCs and CD8^+^ T cells, and facilitating lymphocyte homing; Right side – Chronic STING activation upregulates PD-L1 and IDO-1 expression via the interferon–STAT1 pathway, while inducing IL-10 production that enhances Treg activity and elevating CCL2/7/12 to recruit MDSCs, thereby establishing an immunosuppressive microenvironment and mediating tumor immune evasion. Bottom section highlights the dual implications of cGAS-STING signaling for radiotherapy combined with immunotherapy: on one hand, it converts “cold” tumors into “hot” tumors, enhancing the efficacy of ICIs; on the other hand, it upregulates PD-L1 expression on tumor cells, increasing their sensitivity to PD-L1 inhibitors.

The interferons produced by activation of the cGAS-STING pathway exhibit a dichotomous functionality, where two immune states appear mutually exclusive yet coexist. This connection holds immunological significance because, once a pathogen is contained, the immune system must initiate counter-regulatory measures to prevent immune-mediated pathologies. However, tumor cells can exploit this mechanism through chronic signaling to achieve immune escape (96). Furthermore, from the perspective of current ICIs, the upregulation of PD-L1 expression on tumor cells by radiotherapy enhances their susceptibility to PD-L1 inhibitors, thereby significantly improving the efficacy of anti-tumor immunotherapy through synergistic effects (**Figure 2**).

#### 2.2.3 Radiotherapy upregulates the expression of indoleamine 2,3-dioxygenase 1

Radiotherapy significantly upregulates the expression of indoleamine 2,3-dioxygenase 1 in the tumor microenvironment by activating the IFN-γ-JAK1-STAT1 signaling pathway and the IFN-I downstream NF-κB signaling pathway (97). Specifically, the binding of IFN-γ to its cognate receptor induces tyrosine phosphorylation of STAT1, triggering its dimerization and subsequent binding to the gamma-activated sequence within the IDO1 gene promoter region. Alternatively, tyrosine-phosphorylated STAT-1 acts in concert with NF-κB to induce the transcription of interferon regulatory factor 1 (IRF1); IRF1 then binds to the interferon-stimulated response element (ISRE) in the IDO1 gene promoter. These two signaling pathways ultimately drive the transcriptional activation of IDO1 (98).

IDO1 is a key enzyme in tryptophan metabolism, primarily catalyzing the degradation of tryptophan (Trp) along the kynurenine pathway, converting it into kynurenine (Kyn) (99). This reaction leads to a decrease in local tryptophan concentration, which activates the general control nonderepressible 2 (GCN2) kinase pathway. GCN2 activation subsequently suppresses the proliferation of effector T cells and induces their apoptosis (100). Concurrently, the accumulated kynurenine serves as an endogenous ligand that activates the aryl hydrocarbon receptor (AhR). AhR activation upregulates the expression of interleukin-6 (IL-6), which in turn further stimulates IDO1 expression via the IL-6-dependent STAT-3 signaling pathway, establishing a positive feedback loop (101). IDO^+^ antigen-presenting cells (APCs) directly convert CD4^+^ CD25^-^ T cells into CD4^+^ CD25^+^ Tregs via TGF-β-mediated FoxP3 upregulation, thereby inducing T cell tolerance (102), while simultaneously suppressing natural killer (NK) cell activity (103). Furthermore, *in vitro* experiments have confirmed that IDO1 can also promote tumor cell proliferation by facilitating nuclear accumulation of β-catenin and activating the Wnt/β-catenin pathway (104). MDSCs upregulate IDO expression through IL-6-triggered STAT-3 activation. Concurrently, tumor-derived IDO recruits and activates MDSCs in a Tregs-dependent manner (105). Kynurenine additionally induces DCs to highly express PD-L1 and IL-10, impairing their antigen-presenting capacity (106). Infiltration of IDO^+^ APCs and IDO^+^ MDSCs into the tumor microenvironment and peripheral blood synergistically promotes PD-L1 upregulation and T cell exhaustion, fostering an immunosuppressive tumor microenvironment that drives clinical resistance to radiotherapy combined with immunotherapy.

#### 2.2.4 Radiotherapy acts and potentiates the function of immunosuppressive cells

As mentioned previously, radiotherapy can induce the recruitment of a substantial number of DCs and T cells to the tumor site, thereby promoting anti-tumor immunity. However, immunosuppressive cells may also be attracted by radiation. Radiotherapy can induce tumor cells to secrete various cytokines that attract a variety of immune cells, including M2-type macrophages, MDSCs, and Tregs, among others.

Research has demonstrated that CCL5 can recruit macrophages and promote their polarization into the M2 subtype (107), CCL8 and CCL11 can promote cancer cell proliferation and migration (108, 109). Radiotherapy releases a series of cytokines in the tumor microenvironment through various pathways, including IFN-γ, IL-1β, TNF, IL-4, IL-6, and IL-13.These cytokines primarily promote the differentiation of MDSCs via pathways such as NF-κB (110), STAT1 (111), and STAT6 (112). Concurrently, radiotherapy induces the release of stimulatory factors including Granulocyte-Macrophage Colony-Stimulating Factor (GM-CSF), Granulocyte Colony-Stimulating Factor (G-CSF), and Macrophage Colony-Stimulating Factor (M-CSF), which promote the expansion of MDSCs and their migration into the circulatory system and inflammatory tissues (113). Studies based on ovarian cancer patients have demonstrated that the chemokine CCL22 mediates the trafficking of Tregs to tumors, suppresses tumor-specific T-cell immunity, and promotes tumor growth (114). Furthermore, research indicates that Tregs exhibit greater resistance to radiation compared to other lymphocytes, resulting in their preferential expansion following irradiation (115). In cancer, both Tregs and MDSCs function to negatively regulate immune responses. Tregs secrete inhibitory cytokines, including IL-10, TGF-β, and IL-35, which suppress CD8^+^ T cells and DCs, thereby modulating the host’s anti-tumor immune function (116). Tregs also disrupt cellular metabolism by consuming IL-2 within the tumor microenvironment, thereby inhibiting effector T cells (117). Additionally, Tregs promote adenosine production in the tumor microenvironment through the action of the ectoenzymes CD39 and CD73; the subsequent binding of adenosine to the A2A adenosine receptor (A2AR) on effector cells induces inhibitory and anti-proliferative effects (118). MDSCs express arginase-1 (Arg-1) and inducible nitric oxide synthase (iNOS). Arg-1 promotes tumor progression by degrading L-arginine and, together with the production of reactive oxygen species (ROS), suppresses lymphocyte-mediated immune responses (119). Furthermore, MDSCs can diminish natural killer (NK) cell function by generating inhibitory cytokines, leading to the downregulation of NKG2D expression and reduced IFN-γ secretion (120). Additionally, studies indicate that MDSCs play a direct role in inducing the expression of PD-L1 on tumor cells (121).

Furthermore, factors produced by Tregs and MDSCs can form a positive feedback loop that promotes their mutual expansion and reinforces the immunosuppressive environment. On one hand, MDSCs induce the proliferation of Tregs by generating molecules such as TGF-β, IL-10, CD73, and IDO (122). On the other hand, Tregs modulate the expansion and function of MDSCs through the secretion of IL-35 and TGF-β (123).

**Figure 3:**
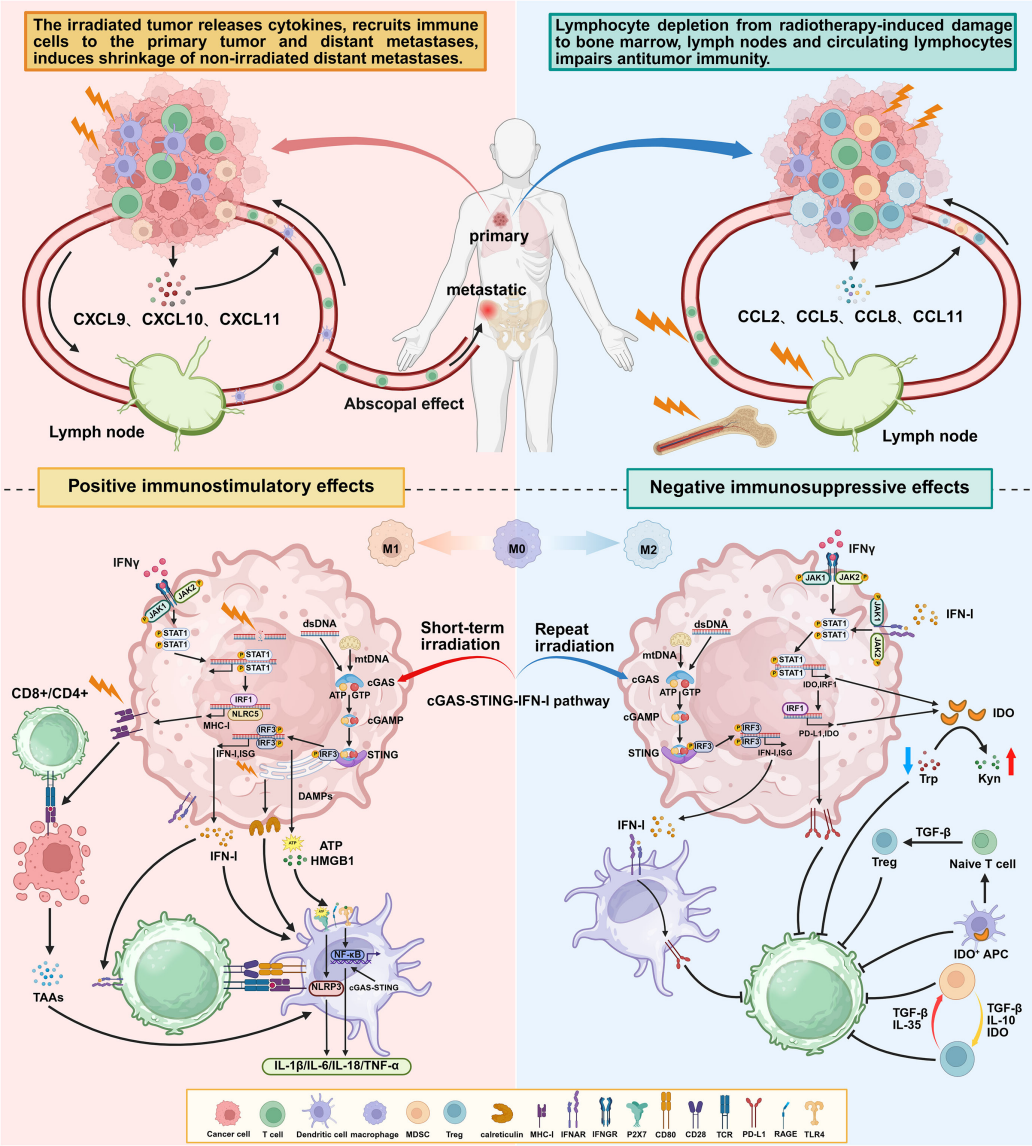
The dual mechanisms of radiotherapy in remodeling the tumor microenvironment. This figure systematically illustrates the dual pathways of immune activation and immunosuppression through which radiotherapy influences immune responses within the tumor microenvironment. The left panel depicts the immunostimulatory effects of radiotherapy: by inducing DNA double-strand breaks and immunogenic cell death in tumor cells, radiotherapy triggers the release of tumor-associated antigens and damage-associated molecular patterns, thereby activating innate immune pathways such as the cGAS–STING–type I interferon signaling axis and NF-κB. This process promotes dendritic cell maturation and effective antigen presentation, leading to the activation of cytotoxic T cells and helper T cells. Concurrently, upregulated MHC-I molecules, exposed calreticulin, and specific chemokine gradients collectively enhance tumor recognition, infiltration, and killing by T cells. Ultimately, these effects may induce abscopal effects targeting distant metastases, establishing a systemic immune-protective response. The right panel illustrates the immunosuppressive effects of radiotherapy: direct damage to the bone marrow, blood vessels, and lymphatic systems can lead to systemic leukopenia and restricted immune responses. Within the tumor microenvironment, repeated irradiation results in chronic activation of the cGAS–STING pathway, shifting it toward a pro-suppressive state, while inducing the production of large amounts of chemokines that recruit suppressive populations such as regulatory T cells, M2-type macrophages, and myeloid-derived suppressor cells. Crucially, radiotherapy upregulates key immune checkpoint molecules, including PD-L1 and indoleamine 2,3-dioxygenase, via interferon signaling, and promotes the conversion of naïve T cells into regulatory T cells through factors such as TGF-β, thereby establishing a robust state of immune tolerance. These suppressive cell populations can further form positive feedback loops, mutually reinforcing each other and synergistically inhibiting anti-tumor immunity.

In conclusion, the ultimate immunomodulatory effect of radiotherapy results from a dynamic interplay between positive immunostimulatory effects and negative immunosuppressive effects. The key to achieving optimal clinical outcomes lies in optimizing radiotherapy strategies—including the selection of appropriate techniques, fractionation regimens, and radiation doses—and rationally combining them with suitable immunotherapy regimens. This approach aims to maximize synergistic anti-tumor efficacy while overcoming or counteracting the associated immunosuppressive side effects (**Figure 3**).

## 3 Immunoprotective effects across diverse radiation therapy techniques

### 3.1 Stereotactic body radiotherapy

Compared to traditional conventionally fractionated radiotherapy (CFRT), SBRT employs a reduced number of treatment fractions (typically 1-5), delivers a high dose per fraction to the tumor, and utilizes image guidance and non-coplanar multi-angle irradiation to provide superior treatment precision. A growing body of evidence in recent years suggests that SBRT may possess unique antitumor immunoprotective effects.

#### 3.1.1 SBRT remodels the tumor immune microenvironment by modulating immune cell subpopulations and activating molecular expression

SBRT not only directly induces tumor cell death but also stimulates local and systemic anti-tumor immune responses by remodeling the tumor immune microenvironment. It enhances the infiltration of immunologically active cells while reducing the population of immunosuppressive cells within the tumor milieu. Specifically, compared to CFRT, SBRT can lead to increases in CD8^+^ and CD4^+^ T lymphocyte counts, elevate the levels of activated NK cell phenotypes, and reduce the populations of Tregs and MDSCs (124).

Navarro et al. conducted immunophenotypic analysis on peripheral blood samples from lung cancer patients treated with SBRT. Flow cytometry revealed a specific increase in CD8^+^ T cells and NK cells in the patients’ peripheral blood, alongside a decrease in the levels of immunosuppressive components, Tregs and MDSCs (125). Zhang et al. demonstrated that following SBRT, the frequency of total T cells in the peripheral blood of NSCLC patients increased, particularly the proportion of CD8^+^ T cells, while the frequency of Tregs decreased (126). In a murine melanoma model, a fractionated regimen employing a moderate radiation dose of 7.5 Gy per fraction provided optimal tumor control and antitumor immunity, concurrently maintaining a lower abundance of Tregs (127). In addition to directly modulating the composition of immune cells, SBRT can also induce the expression of immune-activating molecules and immune-related surface molecules. SBRT enhances the diversity of the T-cell receptor repertoire within the tumor microenvironment, thereby promoting the recognition of tumor-specific antigens by CD8^+^ T cells (128). It upregulates the expression of immunogenic cell surface markers on tumor cells, such as ICAM-1, MHC-I, and Fas (129). Studies involving tumor cell irradiation at doses of 10 Gy or 20 Gy have demonstrated that various cell lines upregulate the expression of Fas, ICAM-1, mucin-1 (MUC-1), carcinoembryonic antigen (CEA), and MHC-I following radiation (130). Furthermore, after SBRT treatment, CD8^+^ T cells express elevated levels of TNF-α, IFN-γ, and IL-2, while concurrently downregulating the production of TGF-β in CD4^+^ T cells (126).

#### 3.1.2 SBRT optimizes dose distribution to reduce circulating blood dose and mitigate radiation-induced lymphopenia

As previously discussed, irradiation of central lymphoid organs (bone marrow and thymus), peripheral lymphoid organs (spleen), and lymphocytes in the blood by ionizing radiation can lead to radiation-induced lymphopenia. Studies have demonstrated that radiation-induced lymphopenia is associated with reduced survival rates in glioblastoma (131), non-small cell lung cancer (132), and pancreatic cancer (133). SBRT reduces the dose to circulating blood by minimizing the target volume and number of fractions, thereby lowering the incidence of radiation-induced lymphopenia. Compared with CFRT, patients treated with SBRT exhibited a lower incidence of severe lymphopenia (134). This phenomenon may be linked to the dose delivered to circulating blood. Dose modeling to circulating lymphocytes indicates that the dose to circulating blood increases with a higher number of treatment fractions. A model developed by Yovino et al. demonstrated that the percentage of blood receiving ≥0.5 Gy increases rapidly with higher total dose and number of fractions. A single fraction of 2 Gy resulted in 4.6% of the total blood volume receiving ≥0.5 Gy. After 10 fractions (20 Gy total), 61.5% of the blood pool was exposed to ≥0.5 Gy, and after 20 fractions (40 Gy total), this proportion rose to 92.2% (135). A study by Crocenzi et al. analyzed peripheral blood samples from 20 patients with pancreatic cancer, comparing a CFRT regimen (50.4 Gy in 28 fractions) with an SBRT regimen (30 Gy in 3 fractions). The results indicated that the hypofractionated radiotherapy schedule avoided the lymphopenia observed with the CFRT regimen (136). What’s more, the target volume in CFRT is typically larger than that in SBRT, resulting in an increased irradiated volume and thereby a higher dose to the circulating blood. Studies in hepatocellular carcinoma have shown that the nadir lymphocyte count during radiotherapy is associated with patient survival. SBRT, with its smaller gross tumor volume (GTV) and fewer fractions, reduces the risk of lymphopenia and has been associated with the highest survival rates (137).

#### 3.1.3 The synergistic effect of SBRT and ICIs in eliciting the abscopal effect

There is growing evidence that ICIs targeting cytotoxic T-lmphocyte-associated protein 4 (CTLA-4) and PD-1/PD-L1 may represent a more effective approach to triggering anti-tumor immunity (138). CTLA-4 is a key negative regulatory receptor on T cells. Studies conducted in mouse models of breast cancer have demonstrated a synergistic effect between CTLA-4 inhibitors and radiotherapy, resulting in a statistically significant survival advantage for the mice; however, CTLA-4 blockade alone was ineffective in controlling tumor growth (139). As mentioned previously, radiotherapy upregulates PD-L1 expression. This mechanism enhances sensitivity to PD-L1 inhibition, significantly improving the efficacy of combining radiotherapy with PD-1/PD-L1 inhibitors. Research indicates that PD-1 inhibitors enhance the antitumor effect of radiotherapy via a cytotoxic T cell-dependent mechanism and synergistically reduce the local accumulation of tumor-infiltrating MDSCs (140). Immune checkpoint inhibitors commonly used in clinical practice currently include PD-1 inhibitors, PD-L1 inhibitors, and CTLA-4 inhibitors (**Table 2**).

**Table 2:** Summary of key information on common immune checkpoint inhibitors.

Mechanisms	Generic names	Initial approval date	Indications
CTLA-4 inhibitor​			
CTLA-4 inhibitors block the binding of CTLA-4 to B7 ligands on APCs, while simultaneously inhibiting the recruitment of phosphatases by the intracellular domain of CTLA-4. This dual action collectively promotes the activation and proliferation of T cells.	Ipilimumab	2011	Melanoma, RCC, HCC, malignant pleural mesothelioma. (with nivolumab)
	Tremelimumab	2022	HCC, NSCLC. (with durvalumab)
PD-1 inhibitor​​			
PD-1 inhibitors specifically bind to the PD-1 on the surface of T cells, preventing its interaction with the PD-L1 on tumor cells.	Pembrolizumab	2014	Melanoma, NSCLC, HNSCC, RCC, classical Hodgkin lymphoma, UC, HCC,gastric cancer, esophageal cancer, cervical cancer, TNBC.
	Nivolumab	2014	Melanoma, NSCLC, HNSCC, RCC, classical Hodgkin lymphoma, UC, HCC, gastric cancer, esophageal cancer, malignant pleural mesothelioma.
	Cemiplimab	2018	Cutaneous squamous cell carcinoma, basal cell carcinoma, NSCLC.
PD-L1 inhibitor​​			
PD-L1 inhibitors specifically bind to the PD-L1 on the surface of tumor cells, preventing its interaction with the PD-1 on T cells.	Atezolizumab	2016	UC, NSCLC, HCC(with bevacizumab), TNBC, melanoma (with cobimetinib + vemurafenib).
	Durvalumab	2017	UC, NSCLC, SCLC, biliary tract cancer (with gemcitabine + cisplatin), endometrial cancer (with carboplatin+ paclitaxel), bladder cancer (with gemcitabine + cisplatin).
	Avelumab	2017	Merkel cell carcinoma, UC, RCC
PD-1/CTLA-4 bispecific inhibitor​​			
​​Simultaneous blockade of PD-1 and CTLA-4​	Cadonilimab	2022	Metastatic cervical cancer

RCC, Renal Cell Carcinoma; HCC, Hepatocellular Carcinoma; NSCLC, Non-small Cell Lung Cancer; HNSCC, Head and Neck Squamous Cell Carcinoma; UC, Urothelial Carcinoma; TNBC, Triple-Negative Breast Cancer; SCLC, Small Cell Lung Cancer.

SBRT not only activates innate immune signaling pathways but also, when combined with immune checkpoint inhibition, can induce adaptive immune responses against both irradiated tumors and distant metastases—a phenomenon known as the abscopal effect. Studies have confirmed that pembrolizumab combined with SBRT induces stronger immune modifications in the tumor microenvironment of non-irradiated sites (141). Furthermore, this combination has demonstrated both efficacy and an acceptable safety profile in non-small cell lung cancer (142), pancreatic cancer (143), metastatic solid tumors (144), and renal cell carcinoma (145). Huang, J. et al. evaluated the induction of systemic anti-tumor immune responses by pembrolizumab combined with SBRT in immunologically “cold” non-small cell lung cancer. The results showed a significant increase in the induction of IFN-γ, IFN-α, and gene sets related to antigen processing and presentation in non-irradiated tumor sites. Significant T-cell clonal expansion was observed in both abscopal sites and the peripheral blood (146). A study in patients with advanced melanoma demonstrated that the combination of ipilimumab and SBRT induced abscopal effect in 52% of the 21 treated patients, and these patients exhibited substantially prolonged overall survival (147). Furthermore, research indicates that hypofractionated radiotherapy (3 × 8 Gy) combined with a CTLA-4 inhibitor can effectively induce anti-tumor immune responses and abscopal effect (148).

### 3.2 FLASH radiotherapy

FLASH RT is a technique that delivers radiation at an ultra-high dose rate (typically exceeding 40 Gy/s), which is three to four orders of magnitude higher than the dose rates used in conventional radiotherapy (0.5–5 Gy/min). Compared to conventional dose-rate irradiation, FLASH RT significantly better protects normal tissues while achieving comparable or superior tumor response. This phenomenon, known as the “FLASH effect,” has been validated across various experimental animal models (e.g., mice, zebrafish, pigs, cats) and in multiple organs (e.g., lung, intestine, brain, skin) (149, 150). Recent studies suggest that FLASH RT may exert unique immunoprotective effects, which could provide a potential explanation for the underlying mechanism of the FLASH effect.

#### 3.2.1 FLASH RT minimizes damage to circulating immune cells through its ultra-short irradiation time

Due to the ultra-high dose rate of FLASH RT, radiation is delivered within an extremely short duration (<0.1 seconds), allowing only a small volume of blood to perfuse the radiation field. In contrast, conventional dose-rate irradiation allows circulating immune cells to continuously enter the irradiated volume via blood flow, leading to more extensive irradiation and killing of these cells. Thus, FLASH RT preserves a far larger fraction of circulating immune cells than conventional radiotherapy.

This preservation effect has been demonstrated in multiple studies. The modeling study by Jin et al. on the impact of different radiation dose rates on circulating immune cell killing demonstrated a strong preservation effect of FLASH RT on circulating blood cells. Specifically, the killing rate of circulating immune cells was reduced from 90%-100% with conventional dose-rate irradiation to merely 5-10% with ultra-high dose-rate FLASH RT. The magnitude of this preservation effect increased with higher doses per fraction, reaching a plateau at 30–50 Gy per fraction, and was almost negligible at a dose of 2 Gy per fraction (151). A model of pencil beam scanning (PBS) FLASH proton therapy demonstrated that, compared to conventional fractionated intensity-modulated proton therapy (IMPT), PBS FLASH proton therapy significantly reduced the number of irradiated circulating immune cells (152). Studies using humanized mouse models of hematopoietic cell transplantation have demonstrated that FLASH irradiation minimizes radiation-induced damage to hematopoietic stem cells and preserves partial functionality of hematopoietic stem and progenitor cells (HSPCs), thereby supporting subsequent hematopoietic regeneration and avoiding both short-term depletion and long-term suppression of circulating immune cells (153).

#### 3.2.2 FLASH RT modulates the immune system and remodels the tumor immune microenvironment

FLASH RT remodels the tumor microenvironment and regulates anti-tumor immunity by modulating key immune cell populations through enhancing lymphocyte infiltration, promoting macrophage M1-like polarization and reducing the proportions of Tregs and MDSCs, indirectly suppressing tumor growth and metastasis; its immunomodulatory effects are comparable or superior to conventional dose-rate radiotherapy, with better normal tissue protection.

Recent studies have demonstrated that FLASH RT can stimulate pro-inflammatory M1-like polarization of macrophages within the tumor microenvironment, both *in vitro* and *in vivo*. This reprogramming enhances the infiltration of both endogenous T cells and adoptively transferred CAR-T cells into the tumor site. The underlying mechanism for this immunostimulatory effect is attributed to the ability of FLASH RT to downregulate the expression of peroxisome proliferator-activated receptor gamma (PPARγ) and Arg-1 (154). Mouse-based FLASH RT studies have demonstrated that, compared to conventional dose-rate irradiation, FLASH inhibits early pro-inflammatory and pro-apoptotic signaling, thereby mitigating radiation-induced acute inflammation and protecting normal tissues. While conventional radiotherapy activates TGF-β-associated pathways that may lead to radiation-induced pulmonary fibrosis, FLASH irradiation downregulates TGF-β levels and effectively suppresses abnormal activation of the TGF-β/SMAD signaling pathway. This suppression inhibits late-stage tumor processes promoted by TGF-β, including metastasis, immune evasion, and angiogenesis (17). Furthermore, the reduction in TGF-β levels inhibits the positive feedback loop between Tregs and MDSCs, ameliorates acute skin toxicity in mice, and attenuates the pro-fibrotic phenotype driven by inflammation and fibroblast activation. Zhu et al. investigated the anti-tumor effects of X-ray FLASH RT versus conventional radiotherapy in a mouse model of breast cancer. At four weeks post-irradiation, the FLASH RT group exhibited a significantly increased ratio of CD8^+^ to CD3^+^ T cells and a markedly decreased ratio of CD4^+^ to CD3^+^ T cells. The results demonstrate that FLASH-RT promotes the influx of CD8^+^ T cells into tumors, reduces the infiltration of macrophages and neutrophils, and mitigates radiation-induced intestinal injury (155).

#### 3.2.3 FLASH RT reduces DNA damage and preserves DNA molecular integrity

FLASH RT preserves genomic integrity via ultra-rapid irradiation, thereby reducing cytoplasmic dsDNA and suppressing the cGAS-STING pathway. Conventional dose-rate irradiation involves dose deposition over hundreds of seconds, causing extensive DNA damage and genomic instability; subsequent irradiation generates excess DNA fragments, which persistently activate the cytoplasmic cGAS-STING pathway and ultimately induce immunosuppression. In contrast, FLASH RT completes dose deposition in 100 ms (nearly instantaneous), precluding the DNA breakage and genomic instability typical of conventional irradiation. This abrogates excess DNA fragment formation, leading to reduction in chronic cGAS-STING pathway activity.

Research indicates that in the context of PD-L1 blockade, FLASH RT is less efficient than conventional dose-rate irradiation at inducing cytoplasmic dsDNA and activating cGAS in intestinal crypts, thereby suppressing CD8^+^ T cell-mediated intestinal pyroptosis (156). *In vitro* studies have indicated that FLASH irradiation reduces the level of DNA damage and leads to attenuated lethality. In mouse lungs, single-cell RNA sequencing and monitoring of proliferating cells revealed that FLASH irradiation minimized the induction of pro-inflammatory genes and reduced the proliferation of progenitor cells following injury. The lungs of FLASH-irradiated mice exhibited less persistent DNA damage and fewer senescent cells (157).

#### 3.2.4 The immunoprotective effects of proton FLASH radiotherapy

Proton FLASH radiotherapy is an emerging technique that delivers highly conformal dose distributions comparable to those of conventional intensity-modulated proton therapy, while simultaneously harnessing the FLASH effect to protect organs at risk surrounding the target volume and conferring potential immunoprotective benefits. Furthermore, comparative studies between proton FLASH and conventional proton therapy have demonstrated significant immunoprotective advantages for the FLASH approach.

Studies have shown that proton FLASH radiotherapy reduces acute skin toxicity and radiation-induced fibrosis in normal tissues while maintaining equivalent tumor control compared to conventional dose-rate proton therapy (158). A study conducted in a NSCLC mouse model demonstrated that, compared to conventional proton therapy, proton FLASH irradiation more effectively reduced tumor burden and suppressed tumor cell proliferation. This was accompanied by an increased infiltration of cytotoxic CD8^+^ T lymphocytes and M1-like macrophages, a decreased proportion of Tregs, and a significant downregulation of PD-L1 expression on tumor cells (159). In a rat glioma model, proton FLASH irradiation provided neuroprotective effects while simultaneously eliciting a robust lymphoid immune response within the tumor, compared to conventional proton therapy (160). Furthermore, studies have shown that lung tumors in mice treated with FLASH were significantly smaller than those treated with conventional dose-rate proton delivery. This was accompanied by an increased recruitment of CD3^+^ T lymphocytes from the peripheral tumor margin into the tumor core. Both CD4^+^ and CD8^+^ T cell populations were increased within the tumor core (161). Proton delivery at FLASH dose rates suppresses the expression of markers associated with radiation-induced inflammation. Specifically, proton FLASH irradiation significantly reduces TGF-β induction compared to proton delivery at conventional dose rates (162). Furthermore, a study by Cunningham et al. observed a trend toward reduced levels of inflammation-related markers following FLASH proton pencil beam scanning. A significant decrease in the levels of CXCL1 and G-CSF was measured in the blood of FLASH RT treated subjects compared to those receiving conventional dose-rate proton pencil beam scanning (163).

### 3.3 Proton therapy and carbon ion radiotherapy

Based on the physical properties of proton and carbon ion beams, the Bragg Peak enables precise energy deposition within the tumor target while minimizing damage to surrounding normal tissues, offering a superior dose distribution compared to photon-based radiotherapy. Furthermore, in terms of RBE, while a value of 1.1 is typically assigned to protons, carbon ions exhibit an RBE ranging between 2 and 5. This higher RBE confers significant advantages to particle therapy over conventional X-rays, including the induction of more lethal complex DNA double-strand breaks, enhanced cell cycle arrest, distinct gene expression profiles, and a reduced impact of tumor heterogeneity. Consequently, particle beams exert a more potent direct killing effect on radioresistant tumor cells and demonstrate stronger immunomodulatory effects.

#### 3.3.1 Proton therapy and CIRT reduce dose to normal organs and circulating blood through the Bragg Peak

Dosimetric analyses of proton therapy and CIRT consistently demonstrate superior dose conformity and enhanced protection of organs at risk (OARs) compared to photon-based radiotherapy across multiple tumor sites (164–166). The improved dose distribution, characterized by a significantly reduced low-dose bath, minimizes irradiation of peri-target lymphoid organs (bone marrow, spleen, lymph nodes) and circulating blood. This physical advantage consequently lowers the incidence of radiation-induced lymphopenia, thereby preserving immune competence during treatment.

Four-dimensional (4D) blood flow models for calculating dose to circulating blood and lymphocytes demonstrate that proton therapy, compared to photon therapy, reduces the mean radiation dose to the blood pool, the maximum dose to the hottest 1% of blood (D1%), and the irradiated blood volume after the first treatment fraction (167). Furthermore, a 4D dynamic liver blood flow model developed by Xing et al. showed that proton therapy significantly decreased both the integral dose and the mean dose to circulating blood for all six patients studied compared to photon therapy. Proton therapy also resulted in lower values for the blood dose-volume histogram (DVH) parameters V>0Gy, V>0.125Gy, and D2% (168). These model-based conclusions are consistent with clinical findings. A study in patients with large hepatocellular carcinoma (>5 cm) indicated that proton therapy reduced the mean spleen dose compared to photon radiotherapy, leading to a significant decrease in the incidence of grade ≥3 lymphopenia (169). Similarly, a comparative analysis of proton versus photon therapy for locally advanced non-small cell lung cancer demonstrated that the reduced irradiated lung volume with proton therapy was associated with a lower rate of severe radiation-induced lymphopenia (170). Clinical evidence demonstrates that CIRT preserves peripheral blood lymphocyte populations (CD3^+^, CD4^+^, CD8^+^ T cells, and NK cells) without significant depletion, indicating minimal lymphotoxicity. This remarkable lymphocyte preservation is fundamentally enabled by the precise dose confinement of the carbon ion Bragg Peak (171).

#### 3.3.2 Proton therapy and CIRT enhance DNA damage in tumor cells and induce immunogenic cell death

High-LET radiation induces clustered DNA lesions that are difficult to repair via common DNA repair pathways (172, 173). The complexity and yield of radiation-induced clustered DNA damage increase with higher ionization density (174). For tumor cells that are resistant to conventional radiotherapy, proton therapy and CIRT can accumulate a greater amount of cytoplasmic DNA, rapidly activate the cGAS-STING signaling pathway, trigger type I interferon transcription, and consequently enhance anti-tumor immunity. Studies using esophageal cancer cell lines have demonstrated that, although proton, carbon ion, and photon radiation can all activate the cGAS-STING signaling pathway through cytoplasmic DNA, distinct differences exist in gene expression responses and biological signaling among these radiation modalities (175). A study using a C57BL/6 mouse tumor model demonstrated that CIRT, at an equivalent RBE, induces more severe and complex DNA damage compared to conventional radiotherapy. This enhanced damage elicits stronger activation of the cGAS-STING pathway, thereby promoting the infiltration of T cells and pro-inflammatory cytokines within the melanoma microenvironment (176). Research on a prostate cancer model demonstrated that following CIRT, the production of cytoplasmic dsDNA, protein levels of p-TBK1 and p-IRF3 in the cGAS-STING pathway, and gene expression levels of downstream interferon-stimulated genes all showed a significant dose-dependent increase. This cascade led to enhanced infiltration of CD4^+^ T cells, CD8^+^ T cells, effector T cells, and macrophages, with CIRT exhibiting more potent tumor growth control compared to photon irradiation (177).

As mentioned earlier, radiotherapy can activate anti-tumor immune responses by triggering ICD in tumor cells, a process associated with the release of DAMPs. Proton and carbon ion beams can more effectively induce the release of various DAMPs, including ATP, HMGB1, and calreticulin (178). Additionally, by causing clustered DNA damage in tumor cells, they induce ICD and synergistically activate the anti-tumor immune response through the release of TAAs and activation of heat shock proteins (HSP70/HSP90). Studies on tumor cell lines have demonstrated that exposure to sublethal doses of proton radiation significantly increases the expression of histocompatibility leukocyte antigens, enhances susceptibility to antigen-specific CTL lysis, and elevates calreticulin expression, leading to increased sensitivity to CTL-mediated killing (179). Research in a melanoma mouse model showed that combined treatment with CIRT and anti-PD-1 therapy more effectively triggers hallmarks of immunogenic cell death—including calreticulin exposure, ATP release, HMGB1 efflux, and induction of type I interferon responses—compared to conventional radioimmunotherapy (180). Furthermore, carbon ion beam irradiation upregulates membrane-associated immunogenic molecules. In locally irradiated tumor tissues from the carbon ion group, ICAM-1 (a marker of DCs activation) remained persistently elevated post-irradiation. Meanwhile, expression of S100A8, a marker of pre-metastatic niche formation in lung tissue, was reduced only in the carbon ion treatment group (181). Studies using the MC38 murine tumor cell line revealed that, at the same physical dose, CIRT exhibits higher cytotoxicity and more pronounced tumor immunomodulation than photon radiotherapy. Compared to photon irradiation, CIRT-treated MC38 cells showed significant upregulation of MHC-I, surface calreticulin expression, and HMGB1 secretion (182).

#### 3.3.3 Proton therapy and CIRT modulate the tumor microenvironment

Proton therapy and CIRT induce phenotypic changes in tumor cells and stromal components, remodeling the immunogenicity of the tumor microenvironment. This remodeling modulates cytokine secretion to recruit immune cells into tumor tissue and reduce immunosuppressive cell populations in the immune microenvironment, collectively enhancing anti-tumor immune function.

Research indicates that proton therapy and CIRT can enhance the infiltration of immune cells such as CD8^+^ T cells, CD4^+^ T cells, and NK cells into the tumor microenvironment. This is accompanied by increased levels of immunostimulatory molecules like IFN-γ, IL-2, and IL-1β, alongside decreased levels of immunosuppressive factors such as IL-10 and TGF-β, thereby inhibiting the infiltration of Tregs and MDSCs (183, 184). A study on hepatocellular carcinoma demonstrated that proton therapy upregulates IL-6 expression in a dose-dependent manner and attenuates the ability of MDSCs to suppress T-cell proliferation (185). In a comparative study between CIRT and photon therapy, CIRT significantly enhanced the secretion of pro-inflammatory cytokines by tumor-infiltrating CD8^+^ T cells, without altering IFN-γ levels. In contrast, high-dose photon therapy only increased IFN-γ secretion by CD8^+^ T cells and promoted the proliferation of Tregs (186). A study by Guo et al. also showed that, compared to X-ray treatment, CIRT induced an increase in CD4^+^ T cells, CD8^+^ T cells, DCs, and NK cells in melanoma (176). Research by Zhou et al. demonstrated that carbon ion beam irradiation reduced the number of MDSCs in the bone marrow, peripheral blood, and spleen of melanoma-bearing mice via a JAK2/STAT3-dependent mechanism, while simultaneously increasing the percentages of CD3^+^, CD4^+^, CD8^+^ T cells, and natural killer cells (187). Similarly, Luo et al. reported that CIRT reduces STAT3 phosphorylation through the JAK2/STAT3 pathway, leading to a decrease in Tregs (188). Compared to conventional low-LET radiation, proton and carbon ion beams not only induce a higher proportion of tumor cell necrosis but also cause more severe DNA damage, leading to enhanced autophagy, apoptosis, and mitotic catastrophe. This cascade results in the release of a greater amount of tumor-specific antigens, generating a more potent immunogenic microenvironment, and ultimately strengthening immune responses against both local and distant tumor lesions.

### 3.4 Spatially fractionated radiotherapy

Spatially Fractionated Radiotherapy achieves tumor control through highly heterogeneous and spatially oscillating dose distributions while minimizing damage to normal tissues. Several preclinical studies using animal models have demonstrated that the highly heterogeneous dose deposition achieved by SFRT is associated with superior immune responses in tumor tissue compared to homogeneous radiation dose delivery. Clinical studies have further shown that SFRT results in reduced toxicity and improved efficacy relative to conventional radiotherapy (189, 190).

#### 3.4.1 SFRT reduces dose to circulating immune cells and protects tumor-draining lymph nodes

SFRT significantly reduces non-specific killing of circulating lymphocytes through its unique physical dose distribution, thereby circumventing radiation-induced lymphopenia. In cases involving long treatment courses, large tumor volumes, and large-field irradiation, conventional radiotherapy indiscriminately damages lymphocytes in the circulatory system. This leads to severe lymphopenia in patients, a condition closely associated with poor prognosis, which undermines the body’s anti-tumor immunity and reduces the response rate to ICIs (191). In SFRT, a large portion of the tumor volume does not receive high-dose irradiation, whereas conventional radiotherapy requires ensuring adequate dose coverage to the entire target volume. By precisely confining high-dose “peaks” to specific vertices within the tumor while allowing most of the tumor volume to receive a lower scattered dose, SFRT significantly reduces the integral dose to the whole body (192). This approach effectively decreases the proportion of circulating lymphocytes exposed to the radiation field during treatment.

Secondly, the treatment strategy of SFRT deliberately avoids delivering high-dose irradiation to TDLNs, thereby preserving these critical hubs for the initiation of adaptive immune responses. TDLNs serve as critical sites where naïve T cells are activated by tumor neoantigens presented by antigen-presenting cells and undergo clonal expansion (193). The inclusion of TDLNs within conventional long-course radiotherapy fields is a significant contributor to therapy-induced immunosuppression (194). This occurs because proliferating T cells are particularly sensitive to radiation damage. When immune checkpoint blockade is administered prior to radiotherapy, it triggers T cell proliferation within TDLNs, thereby rendering these activated CD8^+^ T cells more vulnerable to radiation-induced damage and apoptosis. This chain of events ultimately undermines the abscopal effect of the combined treatment (195). The high-dose peaks in SFRT are deliberately planned to avoid lymph node regions surrounding the tumor. By preserving the integrity of TDLNs, SFRT maintains the immune system’s capacity to initiate anti-tumor immune responses. Studies in breast cancer have demonstrated that, compared to SBRT, mini-grid therapy significantly reduces dose to OARs. Mini-grid therapy achieved a lower mean dose to the ipsilateral lung in all cases while completely avoiding direct irradiation of the contralateral lung. It also resulted in substantial chest wall sparing across all patients, with reduced mean doses to the contralateral breast and spinal cord. Furthermore, it enabled complete sparing of tumor-draining lymph nodes (196). Notably, the quantitative impact of SFRT on circulating blood dose has not yet been fully characterized and requires further investigation.

#### 3.4.2 SFRT remodels the tumor microenvironment through synergistic high/low dose effects

SFRT ablates the microvascular system through its high-dose “peaks,” leading to irreversible DNA damage in tumor cells, ICD, activation of innate inflammatory signaling, and release of TAAs into the tumor microenvironment. Cytokines secreted from these high-dose regions can induce a bystander effect, wherein non-irradiated cells exhibit biological responses due to cytokine signaling from adjacent irradiated cells. This phenomenon may be harnessed to enhance tumor cell killing or protect normal tissues from the detrimental consequences of radiation exposure (197). The potential mechanisms underlying the induction of the bystander effect have been extensively studied in various model systems. These include initiation mediated by gap junction intercellular communication (GJIC), reactive oxygen/nitrogen species, and cytokines such as TNF-α, TGF-β, and IL-8, which activate multiple downstream signaling pathways, including the mitogen-activated protein kinases (MAPKs) and NF-κB pathways (198). This cascade promotes the infiltration of antigen-presenting cells and cytotoxic T cells into the tumor region, thereby altering the tumor immune microenvironment (199). Furthermore, the low-dose “valleys” also exhibit potential immunomodulatory effects. Research on low-dose radiotherapy (LDRT) has shown that LDRT can reprogram the tumor microenvironment and, when combined with immunotherapy, simultaneously induce the mobilization of both innate and adaptive immunity (200). A study using a mouse Lewis lung carcinoma 1 (LLC1) model demonstrated that irradiating 20% and 50% of the tumor volume resulted in the most significant delay in tumor growth. Compared to whole-tumor irradiation, partial-volume irradiation induced a stronger IFN-γ and Th1 immune response while downregulating Th2 immune function. Within the partially irradiated tumor regions, an increase in CD3^+^ cells and TRAIL expression was observed (27). In a separate mouse breast cancer model, MRT was compared with conventional radiotherapy. The results showed that MRT led to a reduction in tumor-associated macrophages and neutrophils compared to conventional radiotherapy, but an increase in T-cell numbers. Furthermore, MRT induced higher levels of pro-inflammatory genes within the tumors than conventional radiotherapy (201).

#### 3.4.3 Combination of SFRT with immunotherapy to elicit the abscopal effect

As previously discussed, SFRT demonstrates the potential to protect organs at risk surrounding the tumor, reduce the dose to circulating blood, and exert immunomodulatory effects. Studies have indicated that when combined with ICIs, SFRT can elicit the abscopal effect, significantly improving treatment outcomes for metastatic cancer (202). It is important to clarify the distinction between the abscopal effect and the bystander effect. The former is defined as the phenomenon where localized radiotherapy to one tumor lesion leads to the shrinkage or disappearance of non-irradiated, distant metastatic lesions. The latter emphasizes contact-mediated or diffusible signaling molecule-mediated communication between cells. While these two effects share some mechanistic similarities, they primarily differ in their site of action. SFRT induces tumor cell necrosis via its high-dose “peaks,” releasing tumor antigens and activating DCs for antigen presentation, thereby initiating a T cell-mediated immune response. This local effect can be amplified by the immune system to target distant tumors. Conclusions from multiple animal studies have demonstrated that SFRT can induce the abscopal effect and exhibits a synergistic relationship with ICIs.

A study conducted in an immunocompetent triple-negative breast cancer mouse model evaluated the efficacy of conventional radiotherapy or SFRT, either alone or in combination with PD-1 and CTLA-4 inhibitors. The results demonstrated that, while conventional radiotherapy combined with immune checkpoint inhibition significantly suppressed tumor growth in the irradiated lesion, it failed to inhibit the growth of distant (contralateral) tumors. In contrast, mice treated with SFRT exhibited evidence of a systemic abscopal immune response, characterized by significantly enhanced infiltration of antigen-presenting cells and activated T cells in the non-irradiated contralateral tumor. This study indicates that applying SFRT to the primary tumor can promote anti-tumor immune responses outside the treatment field, effectively triggering systemic immune activation (203). Another study conducted in a mouse melanoma model compared conventional radiotherapy with MRT, which delivers radiation in sub-millimeter beams separated by non-irradiated volumes. Immunohistological analysis demonstrated that MRT recruits cytotoxic lymphocytes while suppressing Tregs populations and significantly upregulates CXCL9 expression. Furthermore, when combined with anti-CTLA-4 therapy, MRT achieved tumor ablation in half of the cases and induced prolonged systemic anti-tumor immunity (204). These findings suggest that SFRT combined with immunotherapy holds potential benefits for enhancing responses in both local and metastatic disease and appears more effective than conventional radiotherapy in eliciting the abscopal effect.

**Figure 4:**
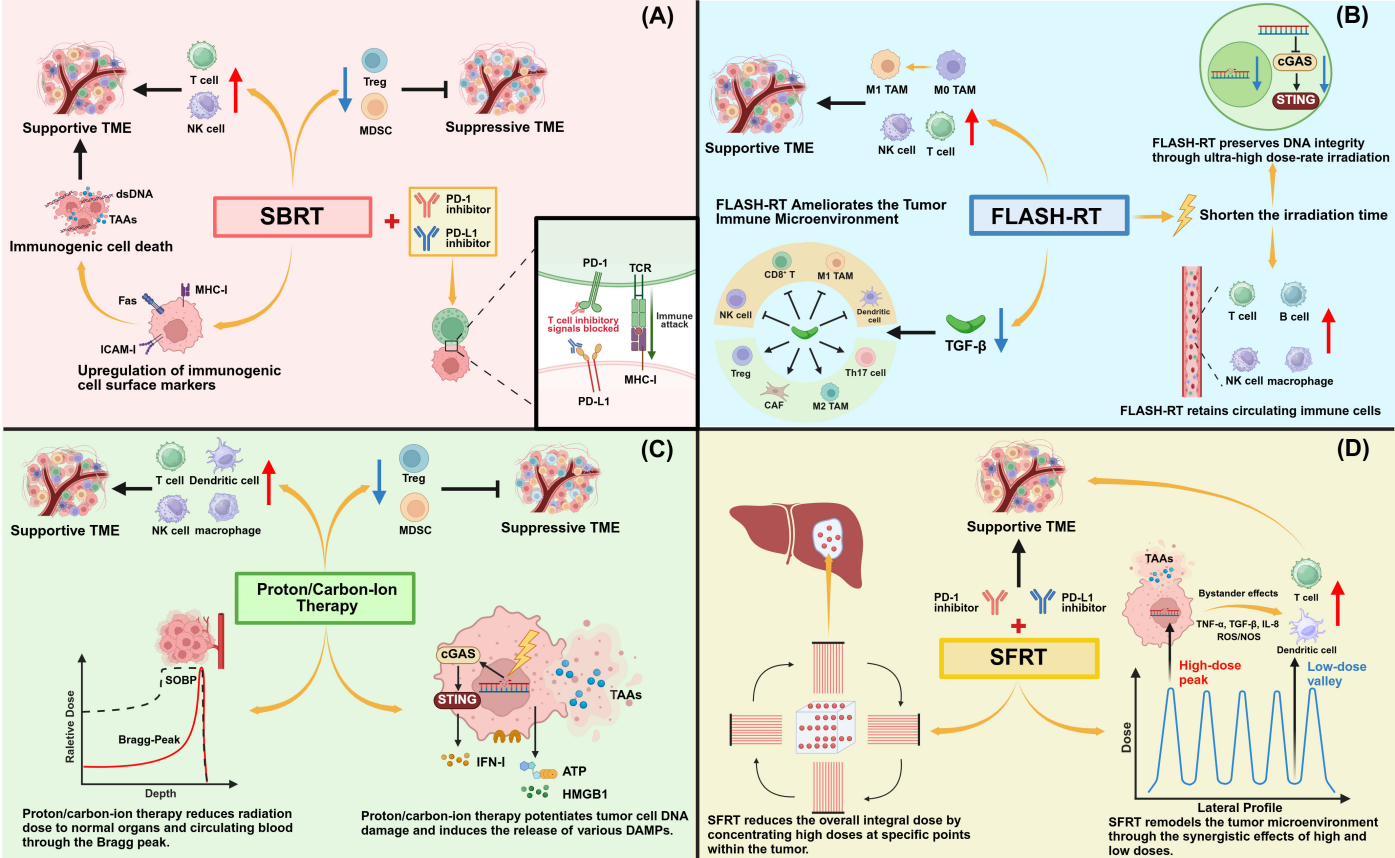
Immunomodulatory mechanisms of different radiotherapy techniques. **(A)** Illustrates the mechanisms by which SBRT upregulates immunogenic cell surface markers, modulates immune cell populations, remodels the tumor microenvironment, and synergizes with ICIs. **(B)** Illustrates the mechanisms by which FLASH RT modulates immune cell populations, reduces TGF-β levels, preserves DNA integrity, and spares circulating immune cells. **(C)** Illustrates the mechanisms by which proton/heavy-ion therapy utilizes the Bragg Peak to protect peri-tumoral tissues, enhance DNA damage, induce immunogenic cell death in tumor cells, and modulate the tumor microenvironment. **(D)** Illustrates the unique dose distribution of SFRT, which reduces the radiation dose to circulating immune cells, synergistically induces bystander effects through the interplay of high and low doses, and remodels the tumor microenvironment.

The aforementioned five radiotherapy techniques are characterized by their core advantage of integrating precise physical targeting and specific biological modulation. On the one hand, they maximize the protection of normal tissues and circulating immune cells through optimized physical dose distribution—such as improving target volume accuracy, leveraging the Bragg peak to achieve sharp dose fall-off, and adopting ultra-high dose rate or spatially heterogeneous irradiation. On the other hand, these unique irradiation modalities elicit prominent radiobiological effects, including the induction of more potent immunogenic cell death, activation of immune pathways such as the cGAS-STING axis, and systematic remodeling of the tumor immune microenvironment. By effectively eradicating tumor cells while minimizing non-specific damage to circulating immune cells and lymphoid tissues, reversing the local immunosuppressive state of tumors, and enhancing systemic anti-tumor immune responses, these techniques provide a crucial scientific basis for maximizing anti-tumor efficacy in combination with immunotherapy and overcoming the immunosuppressive side effects associated with conventional radiotherapy (**Figure 4**).

## 4 A summary of clinical trials on various radiotherapy techniques combined with immunotherapy

In recent years, the combination of photon radiotherapy with ICIs has been extensively studied and its efficacy demonstrated. Particularly, the combination of SBRT with ICIs is a current research focus, with results from several large-scale clinical trials demonstrating excellent safety and efficacy, high rates of pathological response, and the ability to positively modulate the body’s anti-tumor immune response. However, clinical trial data on combining ICIs with techniques such as FLASH RT, proton therapy and CIRT, and SFRT remain limited. Although preclinical studies have indicated potential advantages, further research is needed to confirm these findings.

### 4.1 Stereotactic body radiotherapy

A single-center, randomized, controlled phase II clinical trial (NCT02904954) evaluated the combination of SBRT with the anti-PD-L1 antibody durvalumab in patients with early-stage NSCLC. The results demonstrated that, compared with monotherapy, neoadjuvant durvalumab combined with SBRT significantly improved the major pathological response rate and exhibited a favorable safety profile. These findings indicate that SBRT serves as an effective immunomodulator capable of significantly enhancing the anti-tumor immune response elicited by anti-PD-L1 therapy (205).

A Phase I/Ib clinical trial (NCT03635164) investigating neoadjuvant SBRT combined with a single dose of the anti-PD-L1 antibody durvalumab in patients with HPV-unrelated locally advanced head and neck squamous cell carcinoma (HNSCC) demonstrated a favorable safety profile and encouraging efficacy. Biological correlative analyses revealed a significant increase in CD8^+^ T cells and effector memory T cells within the tumor microenvironment, accompanied by a decreased proportion of Tregs. Enhanced expression of co-stimulatory molecules was observed on DCs. T cell replication was more active in the draining lymph nodes. Significant T-cell receptor clonal expansion was detected in both the tumor microenvironment and the peripheral blood. The peripheral blood also showed an increase in activated T cells and memory T cells, alongside a reduction in immunosuppressive myeloid cells. These results indicate that neoadjuvant SBRT combined with durvalumab is well-tolerated, positively modulates anti-tumor immunity, and induces a high pathological response rate in this patient population (206).

In a randomized, open-label, phase II clinical trial (NCT03071406), the efficacy and safety of nivolumab plus ipilimumab with or without SBRT were evaluated in patients with advanced Merkel cell carcinoma. The study demonstrated that nivolumab combined with ipilimumab, as a first-line treatment, achieved a high objective response rate (ORR) and durable responses with a manageable safety profile in advanced Merkel cell carcinoma. This combination also provided clinical benefit to patients who had previously received anti-PD-1 and PD-L1 therapy. However, the addition of SBRT to this regimen did not further improve efficacy outcomes based on the trial data (207).

A Phase I clinical trial (NCT03223155) evaluated the safety and preliminary efficacy of nivolumab and ipilimumab combined with multisite SBRT in treatment-naïve patients with metastatic stage IV NSCLC. Patients were randomized to either a concurrent treatment group (SBRT administered simultaneously with immunotherapy) or a sequential treatment group (SBRT followed by immunotherapy). Results indicated that concurrent administration of nivolumab, ipilimumab, and SBRT was well-tolerated and demonstrated clinical activity supporting the synergistic effect of SBRT and dual immunotherapy in metastatic NSCLC. However, the sequential treatment group exhibited a higher incidence of toxicities and required dose reductions, prompting the ongoing enrollment for a multi-institutional expansion of the concurrent cohort (208).

A single-arm Phase II clinical trial (NCT02992912) evaluated the efficacy of the anti-PD-L1 antibody atezolizumab in combination with SBRT in patients with advanced colorectal cancer. The results demonstrated that even in the context of radiation-induced lymphopenia, SBRT was able to redirect immune cells toward tumor lesions. A significant increase in intratumoral immune cell infiltration was observed after SBRT, along with elevated PD-L1 expression in the tumors. Furthermore, upregulation of genes such as CCL19, CXCL9, MACF1, and GZMB was detected in the tumor microenvironment; these genes are associated with T/B cell chemotaxis, migration, and tumor cell killing (209) (**Table 3**).

**Table 3:** Summary of clinical trials on SBRT combined with immune checkpoint inhibitors.

Clinical trial ID	Authors	Treated sites (n)	Histology	Type of immune therapy	RT dose/fractions	Control rates	Side effects
NCT02904954	Altorki et al,2021	60 (Group 1 n=30) + (Group 2 n=30)	NSCLC	Group 1: Durvalumab; Group 2: Durvalumab +SBRT.	24Gy/3 Fractions	MPR: Group 1: 6.7% (2/30); Group 2: 53.3% (16/30). ORR: Group 1: 3.3% (1/30); Group 2: 46.7% (14/30).	G3–4 TRAEs: Group 1: 17% (5/30); Group 2: 20% (6/30) G5 TRAEs: Group 1: 6.7% (2/30); Group 2: 6.7% (2/30)
NCT03635164	Darragh et al,2022	21	HNSCC	Preoperative durvalumab + SBRT, surgery and postoperative adjuvant durvalumab	12Gy/2 Fraction (n=3), 18Gy/3 Fractions (n=7), 24Gy/4 Fractions (n=9).	OS Rate: 80.1%. PFS Rate: 75%. MPR or CR: 75%.	Grade ≥3 TRAEs: mucositis oral 19% (the only event occurrence rate exceeding 10%); Grade <3 TRAEs: mucositis oral 66.7%; Dysgeusia 52.4%; Fatigue 57.1%.
NCT03071406	Kim et al,2022	50 (Group A n=25) + (Group B n=25)	Merkel cell carcinoma	Group A: Nivolumab + Ipilimumab; Group B: Nivolumab + Ipilimumab + SBRT.	24Gy/3 Fractions	ORR: Group A: 72.0% (18/25); Group B: 52.2% (12/23); OS: Group A: 29.9; Group B: 16. PFS: Group A: 27.7; Group B: 7.6.	Any Grade TRAEs: 90% G3–4 TRAEs: Group A 40% (10/25); Group B 32% (8/25)
NCT03223155	Bestvina et al,2022	37 (Concurrent Cohort n=18) + (Sequential Cohort n=19)	NSCLC (metastases)	Concurrent Cohort: nivolumab + ipilimumab first and SBRT within 2 weeks; Sequential Cohort: SBRT first and nivolumab + ipilimumab within 1 week.	30Gy/3 Fractions (osseous, spinal and paraspinal metastases); 45Gy/3 Fractions (peripheral lung, liver, abdominal and pelvis); 50Gy/3 Fractions (central lung and mediastinal/cervical or axillary lymph nodes).	ORR: Concurrent Cohort: 44.4% (8/18); Sequential Cohort: 47.4% (9/19). DCR: Concurrent Cohort: 72.2% (13/18); Sequential Cohort: 52.6% (10/19). PFS: Concurrent Cohort: 7.9; Sequential Cohort: 4.7.	G3–4 TRAEs: 62.2% (23/37) G5 TRAEs: 2.7% (1/37)
NCT02992912	Levy et al,2024	60	Colorectal Cancer	Atezolizumab + SBRT.	45 Gy/3 Fractions	PFS: 1.4. OS: 8.4. ORR: 6.8% (4/59).	G3 TRAEs: 5.1% (3/59); G4–5 TRAEs: 0%

ORR, Objective Response Rate; OS, Overall Survival; PFS, Progression Free Survival; TRAEs, Treatment-Related Adverse Events; MPR, Major Pathological Response; DCR, Disease Control Rate.

### 4.2 FLASH radiotherapy

To our knowledge, data on the combined use of FLASH RT and ICIs remain very limited, and this therapeutic approach has not yet been introduced into clinical practice. However, existing preclinical studies have demonstrated its potential benefits. A preclinical study conducted by Eggold et al. (2022) aimed to investigate the potential of abdominopelvic FLASH irradiation combined with PD-1 inhibition in a mouse model of ovarian cancer. The results showed that FLASH RT reduced tumor burden by decreasing intratumoral Tregs and increasing CD8^+^ T cell infiltration, while promoting intestinal regeneration and maintaining tumor control. Compared with conventional radiotherapy, FLASH RT enhanced intratumoral T cell infiltration at early time points and preserved the capacity to potentiate the efficacy of anti-PD-1 therapy. The combination of FLASH and anti-PD-1 inhibitor enhanced tumor control in an anti-PD-1-resistant ovarian cancer model. This study indicates that combining FLASH with ICIs can reduce radiation-induced toxicity, preserve the immunomodulatory properties of radiotherapy, and effectively control tumors, thereby providing evidence for the potential synergistic effect of FLASH RT combined with immunotherapy (210).

### 4.3 Proton therapy and carbon ion radiotherapy

Proton therapy and CIRT represent advanced techniques currently employed in clinical practice. While clinical trial data on the combination of these therapies with ICIs remain limited, several preclinical studies have demonstrated the safety and feasibility of such combinations. Further trials are warranted to comprehensively evaluate their efficacy and optimal application.

A Phase I pilot trial involving patients with unresectable non-small cell lung cancer evaluated the safety, feasibility, and adverse events of durvalumab combined with hypofractionated, dose-escalated, proton beam therapy. Patients received a fixed dose of durvalumab plus an initial hypofractionated proton therapy dose of 60 Gy, with two patients receiving an escalated dose of 69 Gy in 23 fractions. The results showed that all patients experienced treatment-related adverse events, primarily grade 1-2. However, three treatment-related deaths negatively impacted the overall safety profile (211). The study suggests that concurrent durvalumab and hypofractionated proton therapy is well-tolerated in the short term, but its long-term safety requires further evaluation.

A retrospective study of 316 patients with locally advanced non-small cell lung cancer who received concurrent chemoradiation followed by consolidative immune checkpoint inhibition demonstrated that, compared to intensity-modulated radiotherapy (IMRT), proton beam therapy (PBT) significantly reduced the mean heart dose, V15 Gy of the left anterior descending coronary artery, mean lung dose, and the effective dose to circulating immune cells. PBT was also associated with a significant reduction in unplanned hospitalizations within 90 days and the incidence of grade ≥3 lymphopenia (212).These findings indicate that proton therapy not only shows effectiveness in combination with ICIs but also provides superior organ-at-risk protection and reduces the effective dose to circulating cells compared to photon-based radiotherapy.

A multicenter, open-label, non-randomized Phase II clinical trial (NCT05229614) is currently being conducted to evaluate the feasibility of combining CIRT with standard pembrolizumab treatment in cancer patients who have achieved stable disease (SD) following pembrolizumab therapy. Patients will receive irradiation to a single lesion with a total dose of 24 Gy (RBE), delivered in 8 Gy (RBE) per fraction, one fraction per day, over three consecutive days. The primary objective is to assess the clinical response of combining immunotherapy and CIRT across various advanced malignancies in a palliative care setting. Secondary objectives include characterizing the safety profile of CIRT plus pembrolizumab for different advanced malignancies in the palliative setting and evaluating the response of the metastatic lesions treated with CIRT (213).

### 4.4 Spatially fractionated radiotherapy

As previously discussed, numerous studies based on animal models have demonstrated that the combination of SFRT with ICIs can elicit the abscopal effect, thereby improving treatment outcomes in metastatic cancer. However, clinical trial data evaluating the combination of SFRT and ICIs remain insufficient. Nevertheless, several case reports have confirmed its potential efficacy and acceptable safety profile.

A case of multifocal metastatic NSCLC was reported in a patient who had undergone various forms of palliative treatment, including combined chemotherapy and immunotherapy, for multiple metastatic lesions. One metastatic lesion measuring 63.2 cc was treated with high-dose Lattice radiation therapy (HDLRT) in combination with a PD-1 inhibitor. The mass demonstrated a 77.84% reduction in volume within one month post-treatment and achieved a complete remission (CR) after five months, with no treatment-related side effects observed during follow-up examinations. In contrast, none of the other lesions receiving only palliative therapy achieved CR. This marked difference in outcomes underscores the potential efficacy of the combined HDLRT and PD-1 inhibitor approach (214).

A case study involving a patient with widely metastatic melanoma, who had developed resistance to both ipilimumab and pembrolizumab, reported the use of a single 20 Gy fraction using parallel-opposed SFRT combined with ongoing pembrolizumab treatment for a large, painful posterior neck mass. The patient also received a separate course of conventional radiotherapy totaling 50 Gy in 25 fractions. Following five months of combined therapy, the refractory neck mass showed complete resolution with no durable side effects reported (215).

Massaccesi et al. reported a case of a patient with a massive peritoneal metastasis (9.2 × 7.5 cm) from renal cell carcinoma that had progressed following sequential treatment with sunitinib and cabozantinib and was resistant to nivolumab. To alleviate pain and potentially enhance the immune response, the patient subsequently underwent immune-sparing partially ablative irradiation (ISPART). This technique specifically targets only the necrotic core of the tumor, deliberately avoiding doses exceeding 2 Gy to the peritumoral tissue within a 1-centimeter margin to preserve infiltrating lymphocytes in this area. A single fraction of 10 Gy was administered. A follow-up CT scan two months later revealed a 30% reduction in tumor size. The study suggests that ISPART is feasible and exhibits potential synergy with immune checkpoint inhibitor therapy for bulky renal cancer lesions, with its core rationale being the protection of peritumoral immune cells from radiation damage (216) (**Table 4**).

**Table 4:** Comparison of immune cell protection effects, immune-mediated responses, and the Oxford CEBM levels of evidence for different radiotherapy techniques combined with immune checkpoint inhibitors.

Radiotherapy techniques	SBRT	FLASH RT	Proton therapy	Carbon ion therapy	SFRT
Immune cell protection effects ​	SBRT reduces circulating blood irradiation dose via smaller target volume and fewer fractions.	With ultrashort irradiation (<0.1s), FLASH RT minimizes blood flow through the radiation field and reduces killing of circulating immune cells.	The Bragg Peak of proton and carbon ion beams confines high dose to the target volume, sharply reducing irradiation to surrounding normal tissues, peri-target lymphoid organs and circulating blood.		Confining high doses to specific “peaks” exposes most of the tumor volume to low scattered doses.
Immune-mediated responses ​	Modulation of immune cell populations: increase immunocompetent cells (CD8^+^ T cells, NK cells) within the tumor and peripheral blood, while reducing immunosuppressive cells (Tregs, MDSCs).	Modulation of immune cell populations: promoting the infiltration of cytotoxic CD8^+^ T cells and M1-type macrophages, while reducing the Tregs and MDSCs.	cGAS-STING Pathway Activation: induces DNA double-strand breaks, activates the cGAS-STING pathway, and promotes type I interferon production.	Enhanced activation of the cGAS-STING pathway: induces more complex and refractory-to-repair clustered DNA damage, resulting in more robust activation of the cGAS-STING pathway.	Remodeling of the tumor immune microenvironment: high-dose peaks induce ICD, release tumor-associated antigens, activate innate inflammation, and trigger local immunity; low-dose valleys exert immunomodulatory effects, upregulate pro-inflammatory genes, and enhance CD3^+^ T-cell infiltration.
	Activation of immune molecule expression: upregulate immunogenic surface markers of tumor cells (MHC-I, Fas), enhance the diversity of T-cell receptor repertoire, and promote tumor antigen recognition.	Downregulation of key immunosuppressive pathways: suppresses aberrant TGF-β/SMAD signaling activation and downregulates PPARγ and other molecules.	Induction of immunogenic cell death (ICD): effectively induces ICD, releasing DAMPs such as ATP, HMGB1, and calreticulin, and upregulates MHC-I expression.	Induction of immunogenic cell death (ICD): CIRT induces ICD more effectively than proton therapy, and more prominently upregulates the expression of MHC-I and surface calreticulin as well as the secretion of HMGB1.	Bystander effect: through intercellular gap junctions or cytokine (TNF-α, TGF-β) signaling, unirradiated cells are induced to generate biological responses, which amplifies tumor killing or protects normal tissues.
	Abscopal effect: SBRT combined with ICIs elicits adaptive immune responses against primary and distant metastatic lesions.	Maintenance of genomic integrity: ultrafast irradiation decreases DNA damage and extraneous DNA fragment generation.	Remodeling of the tumor immune microenvironment: enhance the infiltration of CD8^+^ T cells, CD4^+^ T cells, NK cells, dendritic cells, and other immune cells within the tumor; elevate immunostimulatory cytokines (IFN-γ, IL-2) while reducing immunosuppressive cytokines (IL-10, TGF-β).	Remodeling of the tumor immune microenvironment: enhance the infiltration of CD8^+^ T cells, CD4^+^ T cells, NK cells, dendritic cells, and other immune cells within the tumor, and significantly reduce the number of MDSCs and Tregs via the JAK2/STAT3 signaling pathway.	Abscopal effect: when combined with ICIs, SFRT induces the abscopal effect more effectively than conventional radiotherapy.
The highest level of Oxford CEBM evidence ​	​​Level 2 (Randomized controlled trials)	Level 5 (Animal experiments)	Level 4 (Phase I pilot trial)	Level 4 (Ongoing multicenter, open-label, non-randomized phase II clinical trial)	Level 4 (Single-case or small-sample case-based analysis)

The highest level of Oxford CEBM evidence are classified based on the OCEBM Levels of Evidence 2 (OCEBM Levels of Evidence Working Group, 2011), available at https://www.cebm.ox.ac.uk/resources/levels-of-evidence/ocebm-levels-of-evidence.

In summary, clinical practice of radiotherapy combined with immunotherapy has achieved substantial progress to date, yet it still faces a series of critical challenges. Among the five radiotherapy techniques discussed, the combination of SBRT with ICIs has demonstrated favorable safety, high rates of pathological response, and positive immunomodulatory effects in multiple clinical trials (205, 206). However, for other advanced radiotherapy techniques such as FLASH RT, proton therapy, CIRT, and SFRT, clinical evidence for their combination with ICIs remains very limited, with most supporting data derived from preclinical studies or small case reports (210, 214–216). Their efficacy, long-term safety, and optimal combination regimens have not yet been fully validated in large-scale randomized controlled trials. Secondly, the efficacy of combining radiotherapy with immunotherapy is influenced by tumor type, the immunotherapy regimen used, and the timing between radiotherapy and immunotherapy. For example, in advanced Merkel cell carcinoma, the addition of SBRT to nivolumab plus ipilimumab did not further improve efficacy outcomes (207); whereas in metastatic NSCLC, concurrent administration of SBRT with dual immunotherapy showed synergistic activity, while sequential treatment was associated with a higher incidence of toxicities (208). This suggests the need for a deeper exploration of the immunological mechanisms underlying the interaction between different radiotherapy techniques and ICIs, in order to more accurately predict and enhance synergistic effects. Meanwhile in the pursuit of therapeutic efficacy, the safety management of combination therapy is of paramount importance, with particular prudence required in terms of dose exploration and patient selection. For instance, treatment-related mortality has been reported in early-phase trials investigating the combination of hypofractionated proton radiotherapy and durvalumab (211), indicating that its long-term safety warrants further evaluation. Finally, there is an urgent need to conduct more well-designed clinical trials for advanced radiotherapy techniques, including FLASH RT, proton therapy, CIRT and SFRT. These trials should span the complete evidence chain, ranging from early-stage proof-of-concept studies to phase III confirmatory trials, and further explore biomarkers predictive of treatment response, so as to ultimately achieve efficient, safe and individualized combination therapy regimens.

## 5 Conclusions

The immunomodulatory effects of radiation therapy have been extensively documented in both preclinical and clinical research. Radiation therapy activates systemic anti-tumor immunity through multiple mechanisms. It can induce immunogenic cell death, release damage-associated molecular patterns, activate DCs to enhance antigen presentation, stimulate the cGAS-STING signaling pathway, upregulate MHC-I expression on tumor cells, and remodel the tumor microenvironment. These immunostimulatory effects can synergize with ICIs, not only potentiating local anti-tumor immunity but also eliciting the abscopal effect, thereby achieving systemic control of metastases outside the irradiated field.

Compared to conventional radiotherapy, the advanced radiotherapy techniques mentioned above demonstrate unique potential immunomodulatory advantages in research. They appear superior to conventional radiotherapy in modulating the immune microenvironment and show promising efficacy and safety profiles when combined with ICIs. By leveraging more precise dose distributions or unique physical properties, these techniques are more conducive to reshaping the immune microenvironment towards a pro-inflammatory, anti-tumor state and may offer a potentially superior therapeutic window for combination with ICIs. Furthermore, these techniques significantly reduce the radiation dose to normal tissues, including circulating blood, lymphatic organs, and organs at risk, through various mechanisms. This is crucial for preserving immune cell function and reducing combination therapy-related toxicity: SBRT achieves precise target coverage through non-coplanar multi-angle beams and real-time image guidance/monitoring; FLASH RT utilizes an ultra-high dose rate and extremely short irradiation time to minimize exposure of circulating blood; proton therapy and CIRT exploit the physical characteristics of the Bragg Peak to provide superior dose conformity and a reduced low-dose bath; SFRT confines high doses to specific, spatially separated points within the tumor, allowing most of the tumor volume to receive a lower scattered dose, thereby substantially reducing the integral dose to the body.

In conclusion, the future development of radiotherapy technology will continue to focus on enhancing delivery precision and deepening integration with immunotherapy. However, clinical data on the combination of immunotherapy with techniques such as FLASH RT, SFRT, and proton therapy and CIRT remain relatively limited. More rigorously designed, large-scale clinical trials are warranted to further evaluate their efficacy and optimal application strategies.

## Glossary

SBRT: stereotactic body radiotherapy
FLASH RT: FLASH radiotherapy
SFRT: spatially fractionated radiotherapy
MHC-I: major histocompatibility complex class I
ICIs: immune checkpoint inhibitors
NSCLC: non-small cell lung cancer
HCC: hepatocellular carcinoma
SRS: stereotactic radiosurgery
CIRT: carbon ion radiotherapy
RBE: relative biological effectiveness
TDLNs: tumor-draining lymph nodes
cGAS: cyclic GMP-AMP synthase
STING: stimulator of interferon genes
dsDNA: double-stranded DNA
mtDNA: mitochondrial DNA
TBK1: TANK-binding kinase 1
IRF3: interferon regulatory factor 3
IFN-I: type I interferons
ISGs: interferon-stimulated genes
DCs: dendritic cells
DAMPs: damage-associated molecular patterns
ICD: immunogenic cell death
CALR: calreticulin
ATP: adenosine triphosphate
HMGB1: high-mobility group box 1 protein
ER: endoplasmic reticulum
TLR4: Toll-like receptor 4
RAGE: receptor for advanced glycation end products
NF-κB: nuclear factor kappa-B
MAPK: mitogen-activated protein kinases
P2X7: P2X purinoceptor 7
NLRP3: NOD-like receptor pyrin domain-containing 3
TAAs: tumor-associated antigens
CXCL: C-X-C motif chemokine ligand
NK cell: natural killer cell
LD_50_: lethal dose 50
LD_90_: lethal dose 90
PD-L1: programmed death-ligand 1
PD-1: programmed cell death protein 1
JAK: Janus kinases
STAT: signal transducer and activator of transcription
IDO1: indoleamine 2,3-dioxygenase 1
Trp: tryptophan
Kyn: kynurenine
GCN2: general control nonderepressible 2
AhR: aryl hydrocarbon receptor
IL: interleukin
Tregs: regulatory T cells
MDSCs: myeloid-derived suppressor cells
Arg-1: arginase-1
iNOS: inducible nitric oxide synthase
ROS: reactive oxygen species
NKG2D: natural killer group 2 member D
A2AR: A2A adenosine receptor
CFRT: conventionally fractionated radiotherapy
ICAM-1: intercellular adhesion molecule-1
CEA: carcinoembryonic antigen
MUC-1: mucin-1
TGF-β: transforming growth factor-beta
GTV: gross tumor volume
CTLA-4: cytotoxic T-lymphocyte-associated protein 4
UHDR: ultra-high dose rate
PBS: pencil beam scanning
IMPT: intensity-modulated proton therapy
HSPCs: hematopoietic stem and progenitor cells
PPARγ: peroxisome proliferator-activated receptor gamma
LET: linear energy transfer
DVH: dose-volume histogram
OARs: organs at risk
HDLRT: high-dose Lattice radiation therapy
CR: complete remission
ISPART: immune-sparing partially ablative irradiation
LLC1: Lewis lung carcinoma 1
MRT: microbeam radiation therapy
3D-LRT: 3D Lattice radiation therapy
MLC: multi-leaf collimator
MC38: murine colon adenocarcinoma 38
HPV: human papillomavirus
ORR: objective response rate
OS: overall survival
PFS: progression free survival
TRAE: treatment-related adverse event
MPR: major pathological response
DCR: disease control rate
UC: urothelial carcinoma
TNBC: triple-negative breast cancer
SCLC: small cell lung cancer
RCC: renal cell carcinoma
HNSCC: head and neck squamous cell carcinoma
